# Long‐term evolution of the Old Rhine estuary: Unravelling effects of changing boundary conditions and inherited landscape

**DOI:** 10.1002/dep2.56

**Published:** 2019-01-02

**Authors:** Tjalling de Haas, Lambertus van der Valk, Kim M. Cohen, Harm Jan Pierik, Steven A. H. Weisscher, Marc P. Hijma, Ad J. F. van der Spek, Maarten G. Kleinhans

**Affiliations:** ^1^ Department of Physical Geography Utrecht University Utrecht The Netherlands; ^2^ Department of Geography Durham University Durham UK; ^3^ Deltares Applied Morphodynamics Delft The Netherlands; ^4^ Deltares Applied Geology and Geophysics Utrecht The Netherlands

**Keywords:** estuary, Holocene, Netherlands, Rhine, river

## Abstract

The long‐term morphodynamic evolution of estuaries depends on a combination of antecedent topography and boundary conditions, including fluvial input, sea‐level change and regional‐landscape interactions. Identifying effects of such boundary conditions on estuary evolution is important to anticipate future changes in specific boundary conditions and for hindcasting with numerical and physical models. A comprehensive synthesis of the evolution of the former Old Rhine estuary is presented here, together with its boundary conditions over its full lifespan from 6,500 to 1,000 cal. yr bp. This system formed during a period of sea‐level high stand, during which the estuary served as the main River Rhine outlet. The estuary went through three stages of evolution: a maturation phase in a wide infilling back‐barrier basin, a stable mature phase and an abandoning phase, both in a laterally confined setting. The Old Rhine River formed by a river avulsion around 6,500 cal. yr bp that connected to a tidal channel within a large back‐barrier basin. Decelerating sea‐level rise caused the back‐barrier basin to silt up around 5,700 cal. yr bp, resulting in shoreline progradation by beach‐barrier formation until *∼*2,000 cal. yr bp. Beach‐barrier formation along the coast and natural levee formation along the river triggered peat formation in the coastal plain, laterally constraining the estuary and limiting overbank deposition, which caused most sediment to accumulate offshore. The abandoning phase started around 2,200 cal. yr bp when a series of upstream avulsions led to a substantial reduction in fluvial input. This induced a period of enhanced estuarine overbank clay deposition that continued into near‐complete silting up and estuary closure around 1200 ad. These findings exemplify how tidal systems, formed in wide coastal plains during sea‐level high stand, depend on antecedent conditions, and how they respond to connection and disconnection of a large river over long, millennial timescales.

## 1 INTRODUCTION

Estuaries are ubiquitous in coastal areas worldwide. They generally form on antecedent topography and substrate and are shaped by changing boundary conditions such as sea‐level rise and independently fluctuating fluvial and coastal sediment supply (Belknap & Kraft, 1985; Coco et al., 2013; Dalrymple, Zaitlin, & Boyd, 1992; De Haas et al., 2018; De Swart & Zimmerman, 2009; Fletcher, Knebel, & Kraft, 1990; Rossi, Amorosi, Sarti, & Potenza, 2011; Seminara, Pittaluga, & Tambroni, 2012; Wang et al., 2012).

Estuaries are important for nature and society. They are very productive natural habitats with a high species density and at the same time are vital areas for agriculture, fishing and ports (Beck et al., 2001; Savenije, 2005). Sustainable use of present‐day estuaries is threatened by changing conditions, such as accelerating sea‐level rise, varying river inflow and increasing human interference with sedimentary processes and ecology (Bouma et al., 2014; Craft et al., 2008; Syvitski et al., 2009; Yang, Wang, Voisin, & Copping, 2015). To adequately anticipate the effects of future changes in boundary conditions, it is important to glean understanding from how such changes have affected estuaries in the past.

While the short‐term effects of changing boundary conditions on estuarine biogeomorphodynamics can be measured and monitored, determining their long‐term (i.e. centuries or more) effects is more challenging. Long‐term estuary evolution can be inferred from geological reconstructions (Allen & Posamentier, 1993; Dalrymple & Choi, 2007; Dalrymple et al., 1992; De Haas et al., 2018; Lessa & Masselink, 1995; Martinius & Van den Berg, 2011) and simulations by numerical and physical models (Guo, van der Wegen, Roelvink, & He, 2014; Kleinhans, Scheltinga, Vegt, & Markies, 2015; Lessa & Masselink, 1995). Each of these approaches requires many simplifications and assumptions. Recent numerical models are able to reproduce realistic width‐averaged estuarine bed profiles reasonably well and thereby provide valuable tools to investigate estuarine sensitivity to changing forcings (Bolla Pittaluga et al., 2015; Canestrelli, Lanzoni, & Fagherazzi, 2014; Guo, Wegen, Wang, Roelvink, & He, 2016; Guo et al., 2014). However, numerical models are not yet able to reproduce self‐confining estuaries in aggrading coastal plains, as they lack the capability to also model critical interactions with the regional landscape and often exclude key biological processes. Moreover, effective hindcasting of estuarine morphodynamic evolution on decadal to millennial timescales requires high‐resolution reconstructions of long‐term estuarine evolution and initial and boundary conditions.

**Figure 1:**
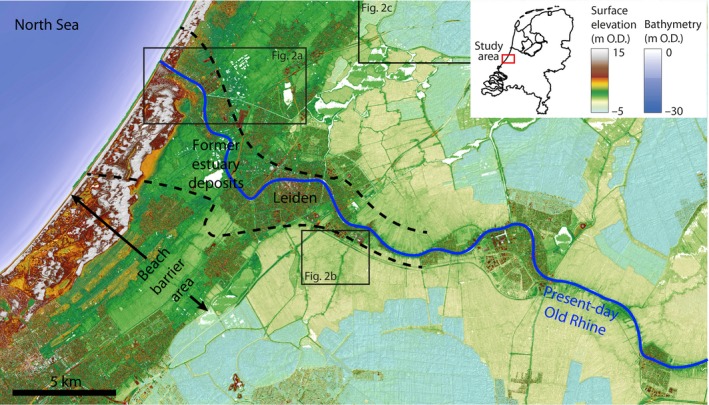
Study area. Extent of former estuary based on Pruissers and De Gans (1988), Van der Valk (1995) and Van Dinter (2013) and geological data in national databases (TNO‐GSN). Elevation data source: AHN (Actueel Hoogtebestand Nederland; Rijkswaterstaat‐AGI, 2005). North is up

**Figure 2:**
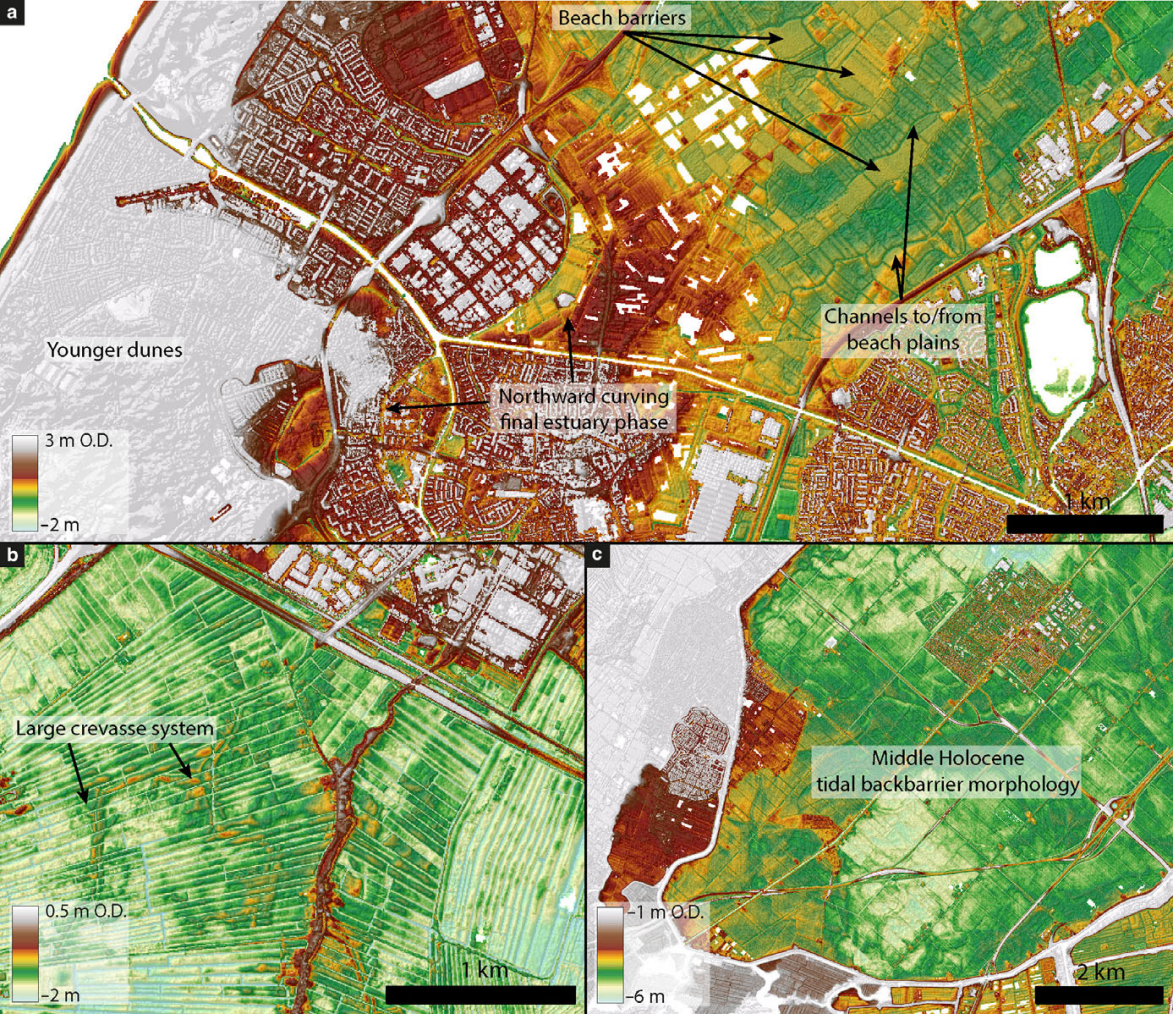
Old Rhine estuary and older tidal back‐barrier deposits expressed in the present‐day surface morphology. (a) Final configuration of the Old Rhine estuary, with a sharp bend towards the north (Van Dinter, 2013). North of the residual channel of the Old Rhine beach‐barrier deposits are exposed. In between the beach barriers the remnants of tidal creeks flowing through the beach plains are present. (b) Large crevasse system in the tidally influenced fluvial part of the Old Rhine. Such crevasse systems are large compared to the crevasse systems in the Rhine delta found further upstream. (c) Middle Holocene back‐barrier basin landscape including many tidal creeks exposed at the surface where overlying peat was mined or eroded by wave erosion in presently drained peat lakes (Haarlemmermeerpolder). See Figure 1 for panel locations. Elevation data source: AHN (Rijkswaterstaat‐AGI, 2005)

Many estuaries inherited from Late Pleistocene valleys incise into bedrock, which controls their position, accommodation and sediment supply (Chaumillon, Tessier, & Reynaud, 2010; Raynal et al., 2010; Rodriguez, Anderson, & Simms, 2005; Vis, Kasse, & Vandenberghe, 2008). The fixed relief in such systems generally inhibits upstream avulsion and loss of fluvial input, which enables long‐term estuary survival (De Haas et al., 2018). In contrast, upstream avulsions are often frequent on wide, low‐relief, coastal and delta plains (Allison, Khan, Goodbred, & Kuehl, 2003; Blum & Roberts, 2012; Edmonds, Hoyal, Sheets, & Slingerland, 2009), inducing large temporal fluctuations in fluvial input to estuaries. As such, river avulsions can reduce or increase the fluvial supply to estuarine river outlets, thereby substantially changing their biomorphodynamics (Lane, Nanson, Vakarelov, Ainsworth, & Dashtgard, 2017). Here, the evolution of the Old Rhine estuary and its changing boundary conditions are described in detail. The Old Rhine was the main river distributary in the Rhine–Meuse delta between *∼*6,500 and *∼*2,000 cal. yr bp (Figures 1 and 2) (Berendsen & Stouthamer, 2000; Cohen, Stouthamer, Pierik, & Geurts, 2012). The Old Rhine estuary formed during a period of sea‐level high stand (Hijma & Cohen, 2011b) and was located in a wide coastal plain. Its boundary conditions have been reconstructed in detail, most notably the timing of river avulsion, which determined the fluvial input to the system. During the Holocene, the Rhine and Meuse rivers entered the same back‐barrier delta in the central Netherlands, wherein most fluvial sediments were trapped before they could reach the open sea (Beets, Van der Valk, & Stive, 1992; Berendsen & Stouthamer, 2000; Cohen et al., 2012). Multiple estuaries became established in the back‐barrier delta plain, connecting river distributaries to the open sea. The Old Rhine River was a particularly long‐lived river distributary, the development of which was strongly affected by major changes in fluvial input as a result of changes in the delta distributary river network as well as in the regional fluvio‐deltaic landscape, which evolved from tidal basin to peat bog over the lifetime of the estuary (Berendsen & Stouthamer, 2000; Hijma & Cohen, 2010, 2011b).

This paper reports on the effects of initial conditions and upstream and downstream boundary conditions on the Old Rhine estuary evolution emphasizing fluvial influx. To this end, the palaeogeographical evolution of the Old Rhine estuary during the period 6,500 to 1,000 cal. yr bp is reconstructed and compared to varying boundary conditions over this period, on the basis of available, partly unpublished, sources and new analyses. New geological cross‐sections of the estuary deposits are presented here. In addition, the spatio‐temporal evolution of the Old Rhine estuary is summarized in a series of newly compiled palaeogeographical maps (Figure 10). These reconstructions reiterate and update past overview studies that cover the Old Rhine estuary (Cohen et al., 2012; Van Dinter, 2013; Vos, 2015) and expand them with the most recent geological and archaeological data. Reconstructed boundary conditions include the avulsive development of the Rhine River network and associated discharge redistribution (Berendsen & Stouthamer, 2000; Cohen et al., 2012; Van Dinter et al., 2017) and the evolution of the coast surrounding the Old Rhine estuary (Pruissers & De Gans, 1988; Van der Valk, 1995), such as the delta front (Van Heteren & Van der Spek, 2008), beach barriers (Beets & Van der Spek, 2000; Cleveringa, 2000) and back‐barrier basin (Beets, De Groot, & Davies, 2003; Beets et al., 1992; Donselaar & Geel, 2007; Vos, 2015).

## 2 GEOGRAPHICAL SETTING AND BOUNDARY CONDITIONS

### 2.1 Geographical setting

The Rhine River is the largest north‐west European river. It is fed by a catchment of *∼*220,000 km^2^, is *∼*1,230 km long, and it has an average discharge of 2,260 m^3^/s and an extreme peak discharge of *∼*12,500 m^3^/s with a return period of *∼*200 years at the Dutch border (Toonen, 2015). The present‐day Rhine River is fully embanked and debouches into the North Sea near the city of Rotterdam in the Netherlands. However, between *∼*6,500 and 2,000 cal. yr bp, most Rhine River discharge followed the Old Rhine River distributary, further to the north (Berendsen & Stouthamer, 2000; Hijma & Cohen, 2011b). The Old Rhine was the delta's main river distributary until Roman times (*∼*2,000 cal. yr bp), functioning as the northern border of the Roman Empire, the *Limes*, in the first to third century ad (Van Dinter, 2013). The present‐day coastal plain of the Netherlands is built up by barrier and back‐barrier deposits on top of a low‐sloping shelf (Beets & Van der Spek, 2000; Vos, 2015).

### 2.2 Offshore boundary conditions

Since the origin of the Old Rhine estuary (Figure 2), *∼*6,500 cal. yr bp, the Dutch coast has been exposed to prevailing westerly winds and dominant south‐west to north‐east‐directed long‐shore drift (Beets & Van der Spek, 2000; Van der Molen & De Swart, 2001a). The current average offshore wave height is *∼*1.5 m, mainly from the south‐west, and storm waves from the south‐west and north‐west may reach up to 6.5 m near the coast (Beets et al., 1992; Jelgersma, Stive, & Van der Valk, 1995; Kleinhans & Grasmeijer, 2006).

**Figure 3:**
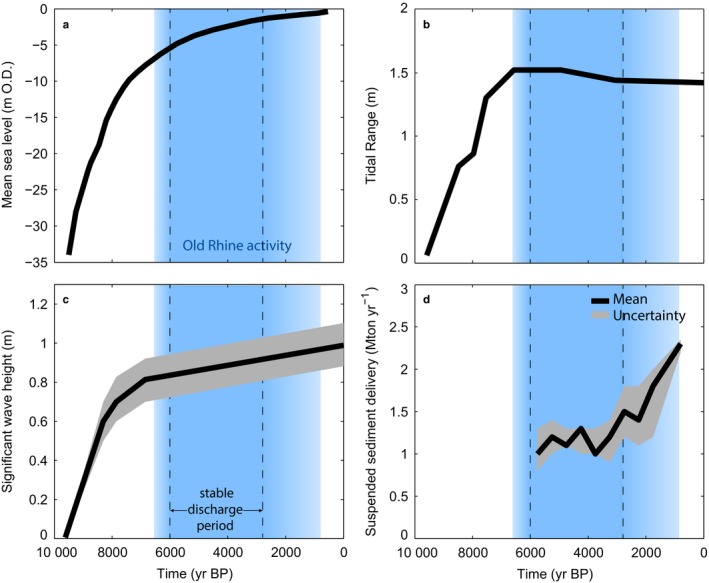
Holocene boundary conditions. (a) Relative sea‐level near the Old Rhine outlet (after Hijma & Cohen, 2010; Jelgersma, 1979; Kiden, 1995; Ludwig, Müller, & Streif, 1981; Van de Plassche, Makaske, Hoek, Konert, & Van der Plicht, 2010). (b) Tidal range near the Old Rhine outlet (after Van der Molen & De Swart, 2001a). (c) Mean significant wave height near the Old Rhine outlet (after Van der Molen & De Swart, 2001b). (d) Trapped part of suspended sediment delivery to the entire fluvial area of Rhine delta (after Erkens, 2009). Period of Old Rhine activity, indicated in blue, is based on Cohen et al. (2012) and Van Dinter et al. (2017). Figure modified from De Haas et al. (2018). All ages in cal. yr bp

The Holocene tidal regime at the mouth of the Old Rhine estuary was microtidal (0.5 to 4 m tidal range along the Dutch coast). Geological observations and modelling studies suggest that the tidal amplitudes have been approximately constant over the last 6,500 cal. yr (Figure 3b) (Hijma & Cohen, 2011a; Roep & Beets, 1988; Van der Molen & De Swart, 2001b), and that net littoral sediment drift has been dominantly northwards since 7,500 to 6,500 cal. yr bp (Hijma, Van der Spek, & Van Heteren, 2010; Van der Molen & Van Dijck, 2000). Middle Holocene mean significant wave height may have been 0.1 to 0.5 m lower than the present‐day wave height due to a smaller North Sea depth as a result of a lower sea‐level (Figure 3c) (Van der Molen & De Swart, 2001a).

Early to middle Holocene relative sea‐level rise was rapid in the Netherlands because of the combined effects of absolute sea‐level rise and accelerated subsidence from forebulge collapse (Figure 3a) (Kiden, Denys, & Johnston, 2002; Vink, Steffen, Reinhardt, & Kaufmann, 2007). From 9,500 to 8,000 cal. yr bp sea‐level rose from −34 to −13.5 m ordnance datum (O.D.; *≈* present‐day mean sea‐level) in front of the Dutch coast (Hijma & Cohen, 2010; Jelgersma, 1979), at an average rate of 1 m per century (Figure 3a). Afterwards, sea‐level rise progressively slowed down from 5.5 mm/year between 8,000 and 7,000 cal. yr bp to a rate of 0.5 mm/year in the last 2,000 years (Hijma & Cohen, 2010; Roep & Beets, 1988; Roep, Van der Valk, & Beets, 1991; Van de Plassche & Roep, 1989).

### 2.3 Variations in total Rhine discharge and sediment delivery

The mean annual discharge of the Rhine was hardly affected by intra‐Holocene climate changes because the size of its catchment is thought to have been largely insensitive to climate. Discharge is estimated to have varied less than 10% during the Holocene (Erkens, 2009; Stouthamer, Cohen, & Gouw, 2011). Deforestation since 3,000 cal. yr bp led to a *∼*60% increase in fine sediment supply by the Rhine River over the period 3,000 to 1,000 cal. yr bp relative to the period 6,500 to 3,000 cal. yr bp (Erkens, 2009; Hoffmann et al., 2007). The frequency of occurrence of large discharge peaks in the Rhine river did vary with intra‐Holocene climate change and growing human impacts (Toonen, 2015; Toonen, Middelkoop, Konijnendijk, Macklin, & Cohen, 2016). The increased flood frequency and fine sediment supply are believed to have led to increased deposition of fines and more frequent avulsions, especially in the last 2,500 years, when the Old Rhine was gradually abandoned (Pierik, Stouthamer, & Cohen, 2017; Van Dinter et al., 2017).

### 2.4 Regional geological evolution

Between 50,000 and 30,000 years ago, the braided Rhine–Meuse river system formed a wide periglacial palaeovalley in the study area (Busschers et al., 2007; Hijma, Cohen, Roebroeks, Westerhoff, & Busschers, 2012). The glacial to interglacial transition caused a climate‐driven transition from fully braided during the Late Pleniglacial to meandering in the Holocene (Berendsen, Hoek, & Schorn, 1995; Pons, 1957). The Late Pleistocene deposits later functioned as the Old Rhine estuary's deeper substrate and as a source for barrier sands from the North Sea floor.

The sea approached the present‐day coast of the Netherlands in the early to middle Holocene, and reached the study area around 8,500 cal. yr bp. This initiated the formation of the modern coastal prism, which is approximately 10 m thick near the Old Rhine estuary and consists of an intercalation of tidal, estuarine and fluvial deposits, including abundant organic beds (Hijma & Cohen, 2011b; Vos, 2015). Before marine drowning and transgression, early to middle Holocene sea‐level rise led to the formation of large wetlands, and resulted in widespread basal‐peat formation on top of the Pleistocene to early Holocene substrate (Beets et al., 1992; Bos, Busschers, & Hoek, 2012; Pons, Jelgersma, Wiggers, & De Jong, 1963; Van de Plassche, 1982). As a result of decelerating sea‐level rise around 8,500 cal. yr bp onwards (Hijma et al., 2010), wave‐driven mobilization of sand in the coastal zone allowed beach‐barrier systems to develop all along the Dutch coast (Beets & Van der Spek, 2000; Van der Molen & Van Dijck, 2000). By *∼*6,000 cal. yr bp, marine and fluvial sedimentation kept up with, or exceeded, the progressively decreasing accommodation rate generated by sea‐level rise, which led to net infilling of the back‐barrier basins (Beets et al., 1992). This induced progressive closure of tidal inlets and beach‐barrier formation, and led to an increasingly water‐logged freshwater environment in the (former) back‐barrier area, resulting in extensive peat formation (Vos, 2015). Only where large rivers debouched into the sea did the tidal inlets remain open, such as at the Old Rhine estuary, whereas tidal inlets not connected to rivers closed by progressive basin infilling (De Haas et al., 2018).

By 3,000 cal. yr bp, most transgressive tidal systems of the western part of Dutch coastal plain were filled up, including the study area. Until *∼*2,000 cal. yr bp the beach barriers and dunes in the western Netherlands gradually accreted seawards, forming in a ∼9 km wide beach‐barrier complex (Cleveringa, 2000; Roep et al., 1991; Van der Valk, 1996). Fed by freshwater (river flood water, groundwater, rain water), peaty marshes, fens and bogs expanded in the back‐barrier area and progressively increased in surface elevation.

## 3 MATERIALS AND METHODS

**Figure 4:**
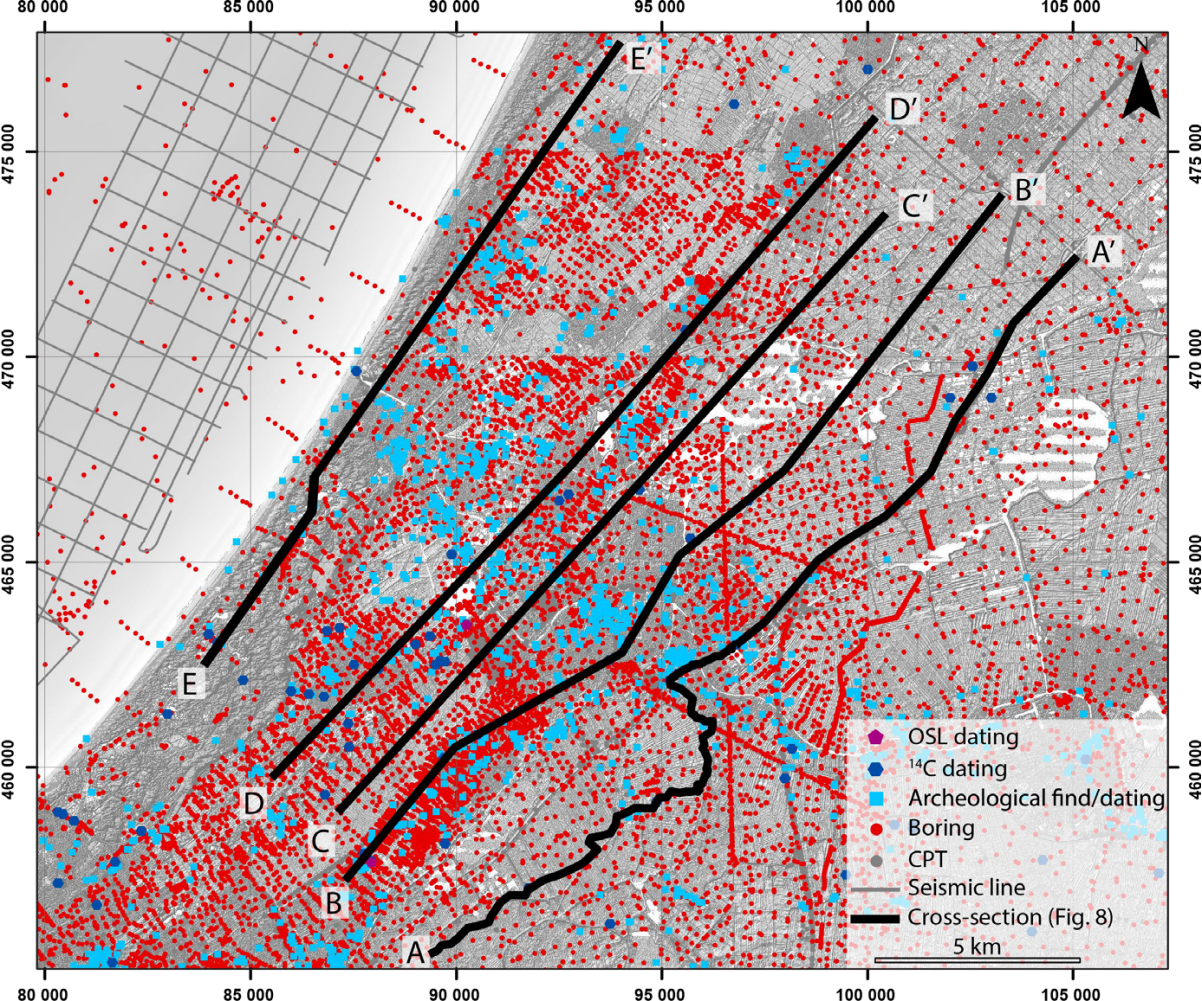
Overview of geological data resources synthesized in this study. Note that multiple dates obtained downcore plot as a single location. Cross‐section locations here given for the geological cross‐sections in Figure 5. Coordinates are in the Dutch national triangulation system (in m). CPT, cone‐penetration test

Geological data from literature and institutional databases were combined to construct cross‐sections and palaeogeographical maps summarizing the spatio‐temporal evolution of the Old Rhine estuary. Changing boundary conditions were independently inferred from the literature on the upstream system and the coastal plain. The geological cross‐sections are based on a new compilation of densely distributed corings and cone‐penetration tests (Figure 4). Borehole descriptions were obtained from the TNO‐DINO database of the Geological Survey of the Netherlands (http://www.dinoloket.nl) and of the UU‐LLG database of the Faculty of Geoscience of Utrecht University (Berendsen, Cohen, & Stouthamer, 2007; Cohen, 2017). Age constraints come from existing radiocarbon (^14^C) (see Supporting Information Tables S1 and S2) and optically stimulated luminescence (OSL) dating, as well as from archaeological finds (Figure 4). Radiocarbon dates were obtained from terrestrial material, particularly peat, and from marine shells (see Supporting Information Tables S1 and S2), while the archaeological finds originate from the Archis 2 national database (http://www.archis.nl).

Five cross‐sections were made across the Old Rhine estuary deposits. Their location was selected such that optimal use of the abundance of bore‐hole descriptions and datings could be made. Cross‐section construction followed established methods for the Rhine–Meuse delta, as documented in Gouw and Erkens (2007) and Hijma, Cohen, Hoffmann, Van der Spek, and Stouthamer (2009) and spans the full thickness of Holocene deposits. Available Holocene ^14^C and OSL dates within *∼*1 km from the cross‐sections were projected on to the cross‐section when the dates belonged to the same geological and lithological unit. High‐resolution laser altimetric (LiDAR) surface‐elevation data with sub‐metre horizontal resolution (Algemeen Hoogtebestand Nederland: AHN; http://www.ahn.nl; Rijkswaterstaat‐AGI, 2005) was also used to capture the surface expressions of sandy channel systems present down to 4 m below the surface because of topographic inversion by differential compaction (Berendsen & Volleberg, 2007). These surface expressions were always verified by borehole data before being applied to the cross‐sections or palaeogeographical maps (cf. Hijma & Cohen, 2011b).

The palaeogeography of the Old Rhine estuary follows from detailed mapping and dating of the distribution and geometry of architectural elements in and between the cross‐sections, each with facies diagnostics of specific depositional environments, following established methods for palaeogeographical reconstruction in the Holland barrier system and Rhine–Meuse back‐barrier delta (Hijma & Cohen, 2011b; Pruissers & De Gans, 1988; Van der Valk, 1995; Vos, 2015). Based on these methods, the following environments of deposition were distinguished, based on their lithofacies, stratigraphy and sedimentology: Tidal basin, tidal channel, estuary/channel, levees/floodplain, wetland (marsh) complex, beach‐barrier complex, dunes, shoreface, delta‐front and Pleistocene substrate (Table 1). The extent of these units on the palaeogeographical maps was determined on the basis of morphological continuity, cross‐cutting relationships and dating evidence (Hijma & Cohen, 2011b; Pierik, Cohen, & Stouthamer, 2016; Pruissers & De Gans, 1988; Van der Valk, 1995; Van Dinter, 2013; Vos, 2015), cross‐checked and corrected with currently available data (corings, ^14^C, OSL and archaeological dates, present‐day topography from LiDAR data) Figure 4).

**Table 1:** Characteristics of the mapped palaeoenvironments

Environments	Lithology	Salinity
Tidal basin	Rhythmic flaser‐bedding and laminated sandy clay, potentially strongly bioturbated	Brackish
Tidal channel	Dominantly sandy, with rhythmic clay layers, coarse gravelly sand beds and predominantly brackish water shells	Brackish
Estuary/channel	Dominantly sandy, with rhythmic clay layers, coarse gravelly sand beds, brackish water shells	Brackish to fresh
Levees/floodplain	Silty clay loam and sandy loam grading into silty clay and humic clay with distance from channels	Fresh to brackish
Wetland (marsh) complex	Peat (mostly reed and forest species) and gyttja	Fresh
Beach‐barrier complex	Fine‐grained and well‐sorted sand, with occasional layers of clay, shells and peat. Locally overlain by coastal dunes with multiple soil horizons	Brackish to saline
Dunes	Fine‐grained and well‐sorted sand, potentially with multiple soil horizons	–
Shoreface	Sandy deposits rich in shell fragments, intercalated with mud layers and marine shells	Saline
Delta‐front	Alternating clay and fining‐upward sand layers, the latter rich in shells	Saline
Pleistocene substrate	Sandy floodplain and aeolian cover sand deposits	Fresh

## 4 OLD RHINE ESTUARY INITIAL CONDITIONS, BOUNDARY CONDITIONS AND RESULTANT STRATIGRAPHY

In this section, the geological data are synthesized to discuss the initial conditions of the back‐barrier basins where the Old Rhine river and estuary formed. The changing boundary conditions of the system are discussed and the stratigraphy and facies of the preserved estuary and delta‐front deposits are described.

### 4.1 Initial conditions

**Figure 5:**
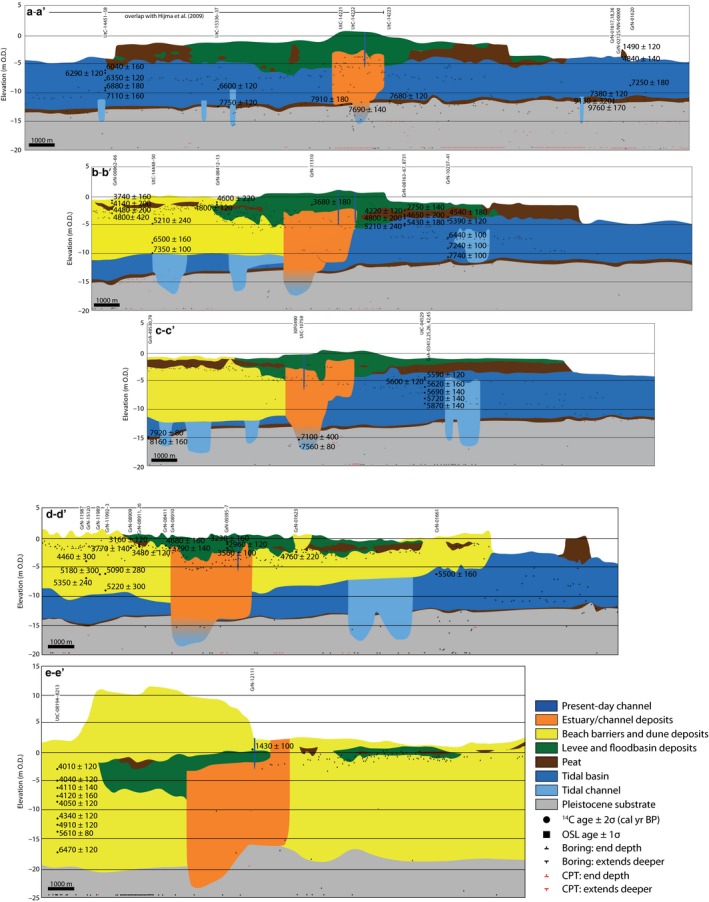
Geological SSW‐NNE cross‐sections of the Old Rhine estuary. See Figure 4 for location of cross‐sections. The southernmost 12 km of profile a–a′ is similar to the cross‐section presented by Hijma et al. (2009). All ages in cal. yr bp. Details on radiocarbon dates can be found in the supplementary tables

The Old Rhine avulsed into a wide back‐barrier basin filled with Holocene tidal deposits on top of a Pleistocene substrate of fluvial and aeolian origin. Near the mouth of the estuary, these pre‐Holocene deposits are found at depths ranging from −11 to −13 m O.D. in the east to about −19 m O.D. in the west (Figure 5). This westward dip in depth of sandy substrate only partly represents the natural 2 *×* 10^*−*4^ m/m pre‐Holocene palaeotopography, because the original surface has been transgressively reworked and eroded by shoreface processes during barrier formation in cross‐section E–E′.

The tidal‐basin deposits are underlain by basal peat up to 1 m thick in cross‐sections A–A′ to D–D′, whereas it is absent in profile E–E′ due to erosion. Radiocarbon dates show that basal‐peat formation started *∼*9,800 cal. yr bp in the areas of lowest relief, expanding broadly and at increasingly higher elevation to become widespread around 9,000 cal. yr bp under the influence of rising groundwaters (Bos et al., 2012; Koster, Stafleu, & Cohen, 2017).

Peat formation stopped around 7,500 to 7,000 cal. yr bp when the area transformed into a tidal back‐barrier basin and conditions became saline. The peat became substantially compressed, as a result of sediment loading, and therefore relatively resistant to erosion. The associated back‐barrier deposits are predominantly clayey at their base, with intercalated humic and sandy layers. Around a depth of −10 m O.D. these deposits generally grade into facies of alternating sand and mud layers, which are predominantly sandy in the proximity of tidal channels and mud dominated at a greater distance. The back‐barrier deposits show that, from c. 7,500 to 5,500 cal. yr bp, the study area went through a sequence of initial drowning with largely subtidal conditions under relatively fast relative sea‐level rise, followed by silting up associated with the formation of intertidal and supratidal areas after the rate of sea‐level rise decreased. This development is in line with the regional evolution of the Holland coast and Rhine delta during this period (Beets & Van der Spek, 2000; Hijma & Cohen, 2011b).

These reconstructions show that, after its formation (6,500 to 6,100 cal. yr bp), the Old Rhine River entered an extensive but largely filled back‐barrier basin, consisting of intertidal and supratidal flats and a well‐developed tidal‐channel network.

### 4.2 Boundary conditions

#### 4.2.1 Fluvial discharge supply

**Figure 6:**
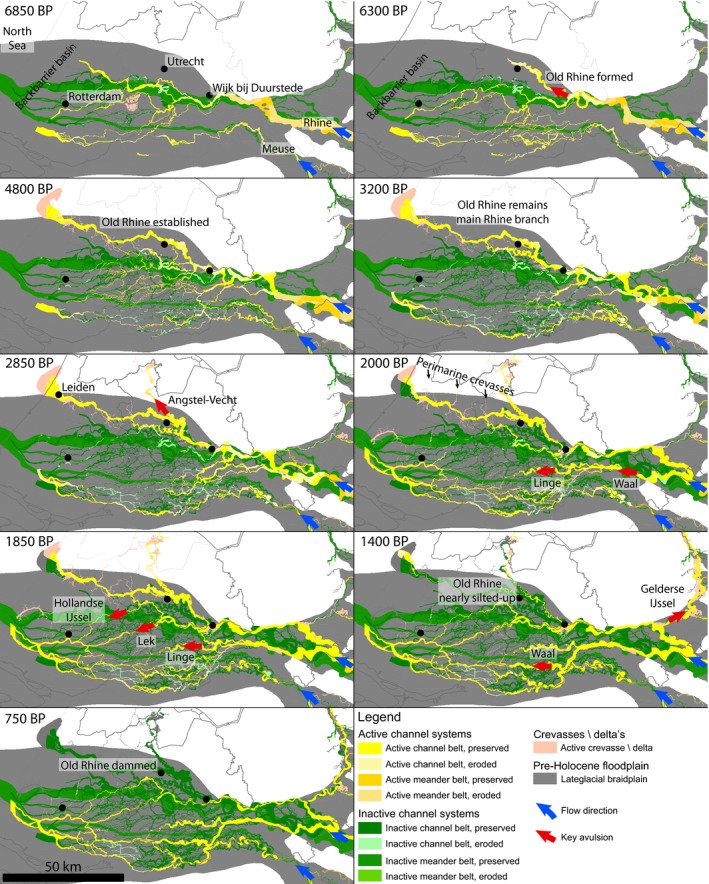
Evolution of the Rhine and Meuse rivers during the lifespan of the Old Rhine, showing initiation (6,300 bp), development, and gradual abandonment (1,850 bp) as new channels increasingly transfer discharge to the southern distributaries in the delta (after Cohen et al., 2012). North is up. All ages in cal. yr bp

Fluvial discharge supply is an important boundary condition for estuary evolution and has strongly varied over time in the Old Rhine River. Around 7,300 cal. yr bp, a first Rhine distributary began to flow into the tidal basin of interest. Around 6,500 cal. yr bp, the next avulsion, 40 km further inland, formed the Old Rhine River distributary (Berendsen, 1982; Berendsen & Stouthamer, 2000; Cohen et al., 2012; Hijma et al., 2009) (Figure 6), which grew to convey the vast majority of the Rhine discharge to the sea around 6100 cal. yr bp, leading to the formation of the Old Rhine estuary. The Old Rhine continued to convey the vast majority of Rhine discharge until *∼*2,200 cal. yr bp (Berendsen & Stouthamer, 2002).

**Figure 7:**
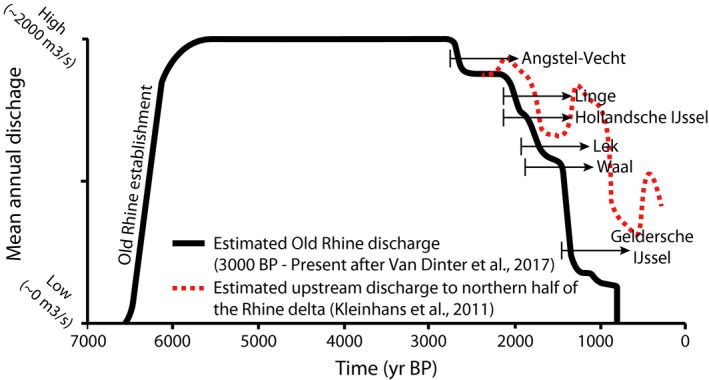
Estimated average discharge in the Old Rhine. Discharge during abandonment and closure of the Old Rhine (2,800 to 800 cal. yr bp) is based on Van Dinter et al. (2017), using upstream channel network configuration, channel dimensions and archaeological finds. Van Dinter et al. (2017) correlate and attribute the loss of discharge to the formation of many new river distributaries in the upstream river network, whereas reconstruction and modelling by Kleinhans et al. (2011) show that changes in the discharge distribution at the Rhine delta apex bifurcation may have partly caused the decrease in discharge through the Old Rhine, where fluctuations arose due to meandering at the bifurcation. All ages in cal. yr bp

Avulsion frequency in the Rhine–Meuse delta peaked between 3,200 and 1,400 cal. yr bp (Berendsen & Stouthamer, 2000; Stouthamer & Berendsen, 2001), which coincides with and may be partly caused by the increased delivery of fine sediment from deforestation (Erkens, 2009; Stouthamer et al., 2011). From 2,850 cal. yr bp onwards, a series of avulsions further upstream in the delta led to a gradual decrease of discharge in the Old Rhine (Figures 6 and 7) (Berendsen & Stouthamer, 2000; Cohen et al., 2012; Kleinhans, Cohen, Hoekstra, & IJmker, 2011; Van Dinter et al., 2017). In addition, changes in the channel and the bifurcation planform shape due to meandering and chute cutoffs at the Rhine delta apex led to a gradual but fluctuating decrease in discharge to the northern part of the Rhine delta and thus the Old Rhine distributary (Figure 7) (Kleinhans et al., 2011). The first avulsion diverting discharge from the Old Rhine was that of the Angstel‐Vecht River (Bos, Feiken, Bunnik, & Schokker, 2009; Cohen et al., 2012; Törnqvist, 1993) (Figure 6), but the resulting discharge loss in the Old Rhine was limited (Figure 7) (Bos et al., 2009; Van Dinter et al., 2017), as inferred from cross‐sectional channel geometries (Van Dinter et al., 2017).

The next series of avulsions, between 2,200 and 1,500 cal. yr bp, at the upstream end of the Old Rhine distributary initiated the new distributaries of the Lek and Hollandse IJssel, leading to a substantial reduction in the discharge of the Old Rhine (Pierik, Stouthamer, Schuring, & Cohen, 2018; Stouthamer & Berendsen, 2001; Van Dinter et al., 2017) (Figure 7). Simultaneously, avulsions further upstream of Wijk bij Duurstede affected the water and sediment supply to the Old Rhine; in particular, the maturation of the Linge, Waal and Gelderse IJssel distributaries (Berendsen & Stouthamer, 2000; Cohen et al., 2012) led to a further reduction of discharge supply to the Old Rhine (Van Dinter et al., 2017). By the end of the 10th century ad the deepest part of the Old Rhine River channel had begun to accumulate a muddy fill, indicating that the channel had become a near‐standing water body. By this time, the Old Rhine at Utrecht carried less water at normal flow than the Angstel‐Vecht and at Wijk bij Duurstede, upstream of Utrecht, much less water entered the Old Rhine than the Lek (Van Dinter et al., 2017). Damming of the Old Rhine at Wijk bij Duurstede in ad 1122 thus merely terminated an already nearly finished process of abandonment (Van Dinter et al., 2017). In short, nearly the full Rhine discharge was conveyed by the Old Rhine between 6,100 and 2,850 cal. yr bp, after which discharge progressively decreased, most rapidly after *∼*2,200 cal. yr bp (Figure 7).

#### 4.2.2 Beach‐barrier development

**Figure 8:**
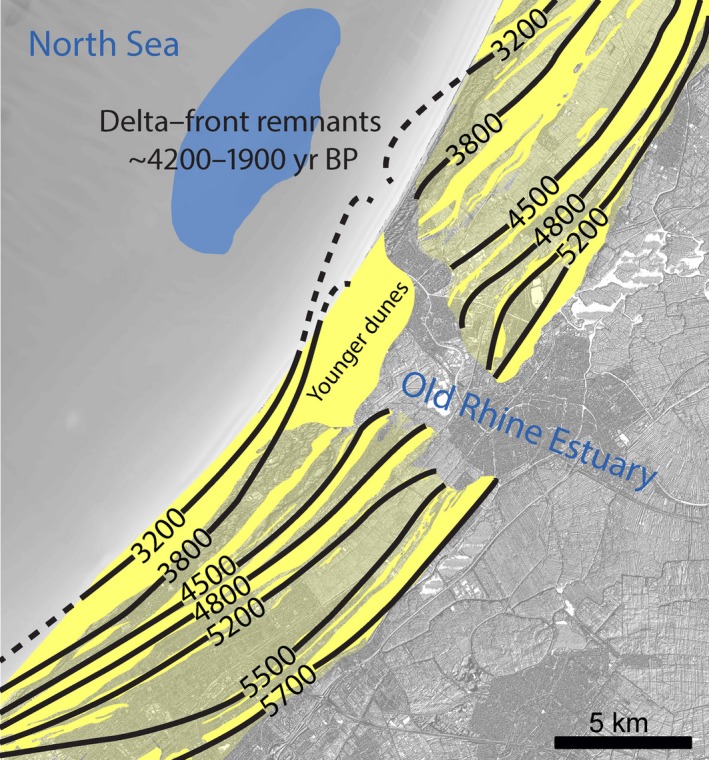
Beach‐barrier sequences (yellow) and preserved delta‐front deposits (blue). Beach‐barrier age after Van der Valk (1995). The beach‐barrier deposits are indicated by a solid yellow fill, while the low‐lying beach plains are indicated by a transparent yellow fill. Preserved distribution of remnant delta front and age range of deposits after Van Heteren and Van der Spek (2008). North is up. All ages in cal. yr bp

At the downstream end, marine processes provided boundary conditions for estuary evolution, predominantly waves that built a set of prograding beach barriers. This resulted in an approximately 9 km wide complex of beach‐barrier deposits along the coast at the Old Rhine outlet (Figures 5 and 8) (Cleveringa, 2000; Roep et al., 1991). The oldest beach barriers flanking the Old Rhine estuary formed around 5,700 cal. yr bp and the youngest preserved beach barriers formed around 2,000 cal. yr bp (Van der Valk, 1995).

The thickness and depth of the base of the beach‐barrier deposits increases seawards. The beach‐barrier deposits become younger from bottom to top and from east to west, in a seaward direction (see dates in cross‐sections B–B′, D–D′ and E–E′ and Figure 8). Beach barriers of the younger part of the barrier system curve seawards near the Old Rhine outlet (Figure 8), showing that the mouth of the Old Rhine was protruding into the sea. This suggests that a substantial amount of sediment was delivered to the sea by the Old Rhine. Especially between 4,800 and 3,800 cal. yr bp there was rapid progradation of the beach barriers, in places exceeding 7 km, corresponding to an average rate of 700 m per century. Beach‐barrier formation differed along the southward and northward sides of the Old Rhine outlet (Cleveringa, 2000; Roep et al., 1991; Van der Valk, 1995). The beach barriers on the south side of the Old Rhine had formed and already prograded by a few kilometres before the first beach barrier on the north side was fully established, which happened around 5,200 cal. yr bp. This observation implies that near‐shore coastal sediment supply from the south was relatively abundant from 5,700 to 5,200 cal. yr bp. This sand supply resulted from erosion of the precursor Rhine–Meuse promontory to the south, which had become established by 7,500 to 6,000 cal. yr bp (Hijma et al., 2010), and was transported to the Old Rhine outlet by the predominantly northward‐directed littoral drift (Hijma & Cohen, 2011b; Hijma et al., 2009). Note that abundant sand was also transported to the beach barriers by cross‐shore transport of shoreface sediments (Van Heteren, Van der Spek, & Van Der Valk, 2011). From approximately 4,800 cal. yr bp onwards, beach‐barrier progradation on the north side of the Old Rhine exceeded progradation in the south. This implies that an increasing amount of sediment was delivered to the coast by the Old Rhine and was subsequently predominantly transported northwards by littoral drift. This is confirmed by the relative abundance of gravel with a Rhine basin provenance admixed with freshwater‐shell fragments north of the Old Rhine outlet compared with quantities on the south side. The distance between the 4,500 and 3,800 cal. yr bp beach barriers on the south side of the Old Rhine progressively decreases with increasing distance from the estuary mouth, suggesting that littoral sand supply decreased during this period.

Between 4,000 and 3,000 cal. yr bp, a large spit formed in front of the Old Rhine estuary, extending from the south, which forced the Old Rhine mouth to migrate northwards (Figure 8). Coastal progradation probably ceased between 2,500 and 2,000 cal. yr bp near the Old Rhine mouth. The exact end time and location of progradation and beach‐barrier formation is hard to pinpoint as the most recent beach barriers were removed by coastal erosion (Heeringen & Van der Valk, 1989; Pruissers & De Gans, 1988; Van der Valk, 2011).

The beach‐barrier evolution shows that the mouth of the Old Rhine estuary progressively migrated in both a seaward and northward direction over time. Moreover, the presence of these beach barriers probably led to relatively tranquil conditions behind them.

#### 4.2.3 Preserved estuary deposits

In the centre of the cross‐sections (Figure 5), estuary channel and bar deposits are present, forming an amalgamated channel belt. The width of the channel and estuary deposits increases in a seaward direction. In cross‐section A–A′, the channel belt has a width of *∼*2 km and reaches down to the basal peat. In its centre, however, the basal peat was eroded and the channel is up to 4 m deeper over a width of nearly 1 km. In cross‐section B–B′, 2 km downstream, the width of the channel‐belt complex increases to almost 3 km. The channel deposits are deepest in the southernmost kilometre of the channel belt, extending through the basal peat and into Pleistocene sandy substrate, whereas the depth of the channel deposits decreases northwards. In cross‐section C–C′, the width of the channel belt is similar to the width of the channel belt in cross‐section B–B′, and the channel depth is also greatest in the southern part of the channel belt. Channel depth rapidly decreases towards the north where the base of the channel is almost 10 m shallower, probably representing a later stage channel of the Old Rhine when the mouth had migrated northwards. Similarly, the channel deposits in cross‐section D–D′ are deepest in the southern part, up to a depth of approximately −17.5 m O.D. (location studied in detail in Busschers et al., 2005, 2007; Törnqvist et al., 2000; Wallinga, Murray, & Bøtter‐Jensen, 2002). The lowest 2.5 m of this sequence are tidal deposits with many brackish water shells and mud layers, showing that these belong to a former tidal channel as shown by Busschers et al. (2007) and in agreement with the hypothesis of Hijma et al. (2009) that the Old Rhine became connected to, and was routed through, a former tidal channel in the early stages of its formation. Near the present‐day coastline, in cross‐section E–E′, the channel or estuary deposits are nearly 4 km wide and here also the deepest deposits are at the southern end of the channel body. Because of lateral migration, the active part of the Old Rhine estuary probably never equalled the eventual envelope width of the estuarine deposits as depicted in the cross‐sections.

**Figure 9:**
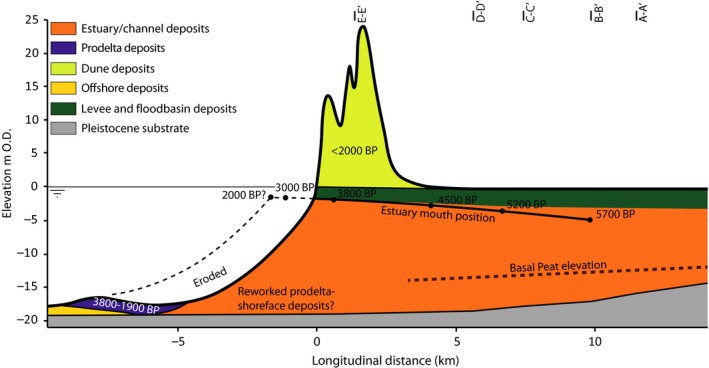
Schematic longitudinal cross‐section of the Old Rhine estuary. The dimensions of the estuary and channel deposits are based on the cross‐sections in Figure 5. Estuary mouth position is inferred from the longitudinal position of the beach barriers combined with sea‐level over time (Figures 3a and 8). The preserved delta‐front deposits are visible in the present‐day bathymetry, and deposit depth is based on Van Heteren and Van der Spek (2008). Basal peat is eroded by the estuary, but the elevation of the basal peat flanking the incised channel of the estuary is denoted by a dashed line. Bathymetry and surface elevation are based on the present‐day surface elevation and bathymetry (AHN; Rijkswaterstaat‐AGI, 2005). All ages in cal. yr bp

The cross‐sections and shallow geological mapping do not allow the location and width of the active channel, or channels, to be directly constrained over time, except for the last stage of the system from 2,500 cal. yr bp onwards (Van Dinter, 2013; Van Dinter et al., 2017). Also, the dimensions and location of the former tidal channel that the Old Rhine connected to are poorly preserved. The maximum depth of the estuary and its channels, however, can be reasonably constrained. Around 6,000 cal. yr bp, the estuary base was at approximately −15 m O.D. in cross‐section A–A′ and lowered to −17 m O.D. in C–C′ (Figure 5). Mean sea‐level at that time stood at approximately −7 m O.D., suggesting an estuary depth of 8 to 10 m (Figures 3 and 9). The channel base at its final functioning stage can be recognized in cross‐section C–C′, at a depth of approximately −8 m O.D. This base is estimated to correspond to the estuary around 2,500 cal. yr bp, when mean sea‐level stood at −1 m O.D., suggesting a local estuary depth of 7 m.

In all cross‐sections, silty to clayey levee, crevasse and floodbasin deposits flank the channel deposits. These silty to clayey Old Rhine deposits wedge out from the main river channel and crevasses towards the surrounding peat. In the seaward cross‐sections these deposits reach for some distance into the beach plains in between the barriers, whereas further landward they are located mainly on top of back‐barrier peats. In the back‐barrier area, flood‐basin deposits laterally grade into peat layers; where the peat contained minimal amounts of clay it has been mined and removed since the 16th century (see the northern parts of cross‐sections A–A′ and D–D′).

In short, the Old Rhine estuary lies within back‐barrier basin deposits grading into beach‐barrier deposits in a seaward direction. The estuarine/channel deposits are incised deepest on their southward side, and the deepest deposits have a tidal‐channel origin showing that the incipient Old Rhine was guided seaward through an older tidal channel. Subsequently, the Old Rhine estuary, in particular its mouth, migrated northwards over time, especially from *∼*4,000 cal. yr bp onwards. The estuary is flanked by levees, which formed when the landscape surrounding the estuary changed from an open tidal back‐barrier basin to a more elongated and confined estuary. At a greater distance from the estuarine channel, great volumes of peat filled the back‐barrier accommodation in the *>*4,000 years that the system functioned.

#### 4.2.4 Offshore delta remains

Delta‐front deposits have been identified in offshore seismic profiles and cores by Van Heteren and Van der Spek (2008) (Figure 8). They are found up to approximately 8 km seawards of the present‐day coastline at −20 m O.D., located in a 10 *×* 5 km zone, with the widest axis parallel to the coastline. The patch of delta‐front deposits is lens‐shaped, relatively thin (2 m thick), and comprises a sequence of alternating clay and sand layers, the latter rich in shells. The shells are concentrated at the base of the sand layers, suggesting fining‐upward sorting during deposition. ^14^C dates collected from the preserved sediments show that they were deposited between 4,200 and 1,900 cal. yr bp, and that most accumulation took place between 3,800 and 3,300 cal. yr bp (Cleveringa, 2000; Van Heteren & Van der Spek, 2008). This coincides with the fast beach‐barrier progradation occurring during this period, while the age of the youngest deposits coincides with a period of substantially decreasing river discharge. Older delta‐front deposits (i.e., from before 4000 cal. yr bp) appear to have been buried and eroded by shoreface processes during coastal transgression.

## 5 SYNTHESIS AND DISCUSSION

**Figure 10:**
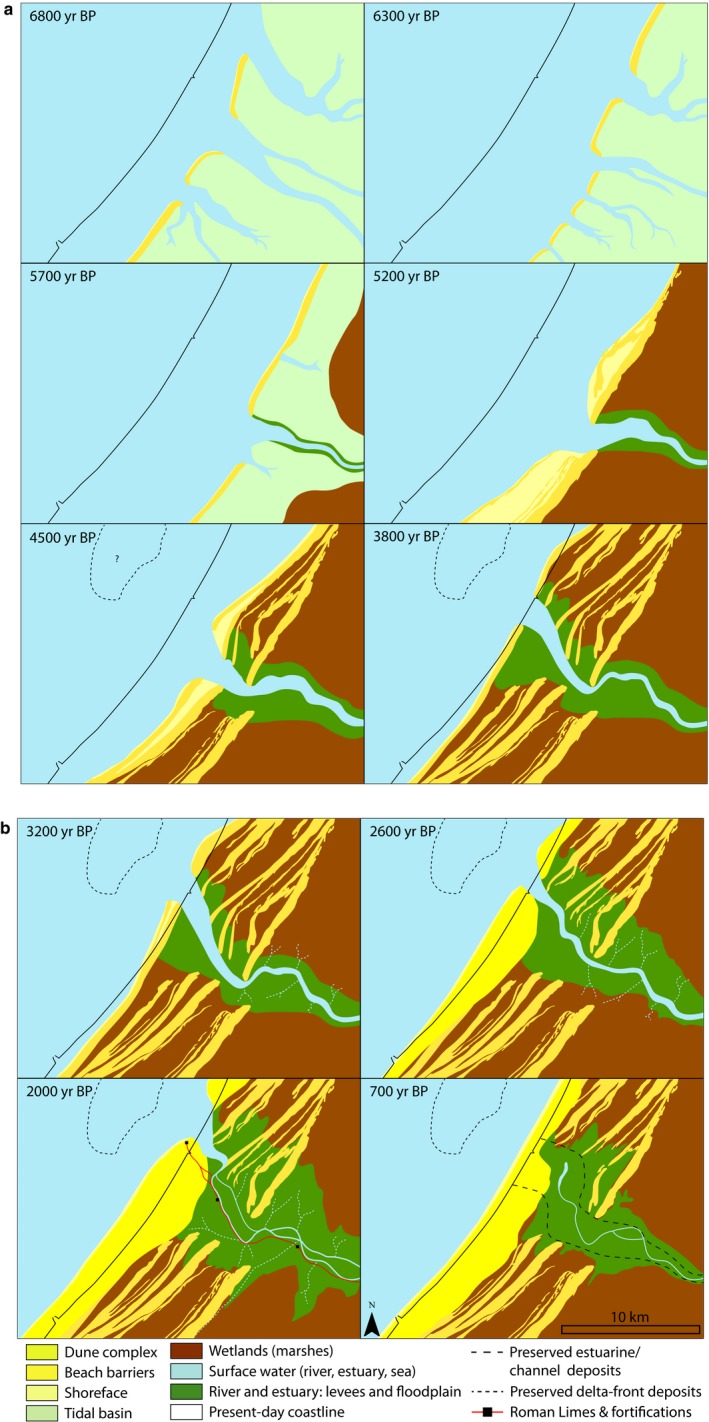
Palaeogeographical reconstruction of the evolution of the Old Rhine estuary, based on the data presented here and maps presented by Pruissers and De Gans (1988), Van der Valk (1995), Hijma and Cohen (2011b) (6,800 and 6,300 cal. yr bp) and Van Dinter (2013) (2,000 cal. yr bp). The exact channel width over time cannot be determined with certainty based on the presently available data for the period 6,800 to 2,600 cal. yr bp, and is estimated here. All ages in cal. yr bp

Here the spatio‐temporal evolution of the Old Rhine estuary is synthesized into a series of palaeogeographical maps of the study area from 6,800 bp to 700 bp (Figure 10). These maps are used to discuss the long‐term morphodynamic evolution of the estuary in time and space. Separate phases of ‘establishment’ (6,800 to 5,700 cal. yr bp), ‘progradation’ (5,700 to 2,000 cal. yr bp) and ‘abandonment and closure’ (2,000 to 800 cal. yr bp) are distinguished. The estuarine morphodynamic developments for each of these phases have different spatial scales: the hydromorphologically active area for estuarine developments was much greater in the first phase than in the later phases.

### 5.1 Establishment of the Old Rhine estuary

The 7,300 to 5,700 cal. yr bp time frame was characterized by the establishment of the barriers and tidal basin (Figure 10: 6,800 to 5,700 cal. yr bp). In terms of the development of the Old Rhine estuary, however, it can be seen as the establishment of the initial conditions. These initial conditions are distinct from many other estuaries in the world. Firstly, because of the wide back‐barrier plain along the Dutch coast, the Old Rhine could freely move away from former estuaries, connecting to and subsequently capturing a tidal channel outside the formerly active part of the delta, which consequently expanded in size (Figures 5 and 10). This was possible because sea‐level rise had exceeded the palaeovalley shoulders, creating a broad plain of accommodation, unlike the situation in topographically constrained estuaries elsewhere (Allen, 1990; Belknap & Kraft, 1985; Bertin, Chaumillon, Weber, & Tesson, 2004; Clement & Fuller, 2018; Fletcher et al., 1990; Gregoire, Le Roy, Ehrhold, Jouet, & Garlan, 2017; Long, Scaife, & Edwards, 2000; Pye & Blott, 2014; Vis et al., 2008, 2016). Secondly, the Old Rhine entered an infilling tidal basin with an abundant intertidal area and a well‐developed tidal‐channel network, through which it could rapidly obtain a connection to the open sea (Figure 10: 6,800 to 5,700 cal. yr bp). Finally, the gently sloping, sandy continental shelf in the southern North Sea enabled rapid transgression of the tidal basins along the Holland coast (Beets & Van der Spek, 2000). Furthermore, a considerable amount of sand was available for waves to build wide beach barriers and to fill the back‐barrier basins in combination with fluvial mud. This contrasts with the numerous tidal systems with a steeply sloping Pleistocene surface and relatively small sediment influx (Takashimizu et al., 2016), which filled in slowly as rivers formed bay‐head deltas of coarse material. Returning to the Old Rhine estuary, the initial tidal basin configuration into which the Old Rhine avulsed not only determined the location of the new river, but it also allowed the newly avulsed channel to rapidly establish a direct connection through an existing tidal inlet to the sea rather than diverge into the back‐barrier basin. So, the combined processes of upstream avulsion and downstream tidal‐channel piracy determined the position of the Old Rhine river for more than 4,000 years. In addition, the limited accommodation in the tidal basin during the time of formation of the Old Rhine distributary enabled rapid throughflow of fluvial sediment to the coast, enabling coastal transgression. Moreover, it inhibited the formation of a bay‐head delta, in contrast to many other rivers entering tidal basins (Boyd, Dalrymple, & Zaitlin, 1992; Dalrymple et al., 1992; Padmalal et al., 2013).

In short, transformation from a tidal basin to an estuary in the study area initiated between 6,500 and 5,700 cal. yr bp, when the Rhine avulsed into the tidal basin and the barrier system stabilized its position. Due to the decreasing rate of sea‐level rise and an increase in discharge in the Old Rhine, a well‐developed river channel was established through the former back‐barrier basin around 5,700 cal. yr bp, which also started to develop levees that laterally confined the main water flow (Figure 10: 5,700 cal. yr bp). Progressive levee growth isolated the back‐barrier area from the estuary, which facilitated peat development at rates sufficient to keep up with the decelerating sea‐level rise. The peat development restricted channel migration, thereby keeping the levees in a relatively stable position and enabling vertical levee growth. Whether this was of prime importance for the development of the estuary, merely accelerated its development or had no significant effects, cannot be determined based on the available data.

The above developments are unique to the surviving river outlet of the Old Rhine estuary. All surrounding Middle Holocene tidal inlets show the opposite development of silting up and closure, followed by transformation into vast back‐barrier peat lands.

### 5.2 Coastal progradation and deltaic outlet phase

Progressive closure of other tidal inlets along the barrier coast formed relatively protected environments at the inland side, but on the other hand initiated erosion of the ebb‐tidal deltas (Beets & Van der Spek, 2000; De Haas et al., 2018; Roep et al., 1991). In combination with the large volumes of available sand on the shallow North Sea floor, this resulted in a surplus of sandy sediment supply to the coast and its shoreface, leading to coastal progradation (Figures 8 and 10) (Cleveringa, 2000; Hijma et al., 2010; Roep et al., 1991; Van Heteren et al., 2011). Silting up of the back‐barrier basin caused the distribution pattern of marine sediment to change from cross‐shore redistribution, in which sediment entered the back‐barrier basin via the tidal inlets, to alongshore redistribution, leading to new barrier formation seawards of older coastlines. Moreover, the Old Rhine was now established as an estuarine channel, efficiently bypassing its increasingly filled tidal basin and hence supplying substantial parts of its fluvial water and sediment fluxes to the seaward side of the coastal barrier system. This resulted in the formation of a cuspate river mouth flanked by beach barriers. Between 4,000 and 3,500 cal. yr bp, an increasing amount of sand was incorporated into the beach barriers flanking the Old Rhine outlet, especially on its northern side (Figure 8). Accordingly, delta‐front accumulation rates were relatively high during this period (Van Heteren & Van der Spek, 2008). Around the same time, a large spit formed in front of the southern edge of the Old Rhine River mouth, partly blocking the estuary inlet and forcing the Old Rhine River mouth northwards (Figure 10: 3,800 to 3,200 cal. yr bp). From 3,500 to 3,000 cal. yr bp, the coastal progradation rate decreased, which was probably the result of sediment depletion off the coast caused by progressive steepening of the shoreface (Beets et al., 1992; Van Heteren et al., 2011).

Between 5,700 and 3,000 cal. yr bp, the Old Rhine discharge was relatively stable (Figure 7). In this period sediments originating from the river and estuary settled from suspension on the prograding delta front. In addition, mud was trapped in the estuary, in the beach plains surrounding the estuary and at a relatively small scale along the Old Rhine River channel. There was extensive peat formation inland of the beach barriers and on a smaller scale between the inner beach barriers. The peat‐land environment implies that fresh groundwater level was high; freshwater lenses had formed in the top of the beach‐barrier complex aquifer and in the back‐barrier deposits (Delsman et al., 2014). Also, mud supply was low to absent as a result of established levees and dense riparian vegetation, both separating the main channels from the flood basins and causing initiation of raised‐bog formation in the most distal areas (Pierik, Cohen, Vos, van der Spek, & Stouthamer, 2017; Van Dinter, 2013; Vos, 2015). The accumulated peat layer aided in hampering lateral meander migration (cf. Makaske, Berendsen, & Van Ree, 2007) and caused relatively high and narrow levees (Van Asselen, 2011). In addition, the peat filled in considerable accommodation space in the back‐barrier area from approximately 5,700 cal. yr bp, which restricted lateral sediment storage and caused fluvial sediment to more efficiently bypass to the coast. This bypass is observed in the expansion of the beach barriers and the development and outbuilding of the delta front.

The expansion of the spit from the southern bank of the estuary mouth around 4,000 to 3,500 cal. yr bp (Figure 10: 3,800 cal. yr bp) led to relatively sheltered conditions in the floodplain behind it, resulting in the deposition of marine and fluvial muds (see cross‐section E–E′ in Figure 5), particularly in the south‐western part of the estuary. Especially in the seaward parts of this mud bed, marine diatoms and molluscs indicate deposition under saline conditions (Van der Valk, 1995).

Palaeo‐ecological studies indicate that saline storm surge water occasionally reached as much as 15 km upstream from the estuary mouth during Roman times (Van Dinter, 2013). Blocking of river discharge during storm surges caused the water level in the lower reach of the river to rise, which caused overtopping of the levees and allowed ‘perimarine’ crevasse channels to branch off to be reused in subsequent events, helped by tidal currents as shown by tidal bundling in the sediment infills. These crevasse channels have a land inward orientation and occur abundantly between 10 and 20 km upstream of the present coastline from where they extend into peat lands. It is estimated that these crevasses started forming from 4,000 to 3,500 cal. yr bp onwards, when the fluvio‐tidal levees along the main system became more mature (Berendsen, 1982; Cohen et al., 2012; Van Dinter, 2013) (Figures 2b and 10: 3,200 to 2,000 cal. yr bp). From 20 to *∼*30 km inland, a decrease in abundance and size of these crevasse channels is observed. This suggests that the tidal backwater effect reached up to at least *∼*30 km inland (Martinius & Van den Berg, 2011; Van Dinter, 2013), which agrees with a characteristic backwater length estimated as water depth, here about 10 m, divided by gradient, here about 0.5 *× *10^*−*4 ^m/m to 1 *× *10^*−*4 ^m/m. Along the Old Rhine, the perimarine crevasses were most widely established around 3,000 cal. yr bp (Berendsen, 1982; Cohen et al., 2012). At this stage, the matured crevasse systems functioned as year‐round drainage channels for the peat lands, with semi‐diurnal tidal currents preventing the channels from silting up or paludifying. During storm surges and river peak discharge, they would still temporarily develop landward gradients and function as crevasse channels.

### 5.3 Loss of discharge and estuary closure

Discharge from the Old Rhine started to drop from 2,850 cal. yr bp (Figures 6 and 7). The loss of discharge was most pronounced between 2,200 and 1,500 cal. yr bp, when the Hollandse IJssel and Lek avulsions grew into new permanent channels (Berendsen & Stouthamer, 2000; Cohen et al., 2012; Pierik et al., 2018). The decrease in river discharge coincided with a period of enhanced marine clay deposition in the Old Rhine estuary (Figure 10: 2,600 to 2,000 cal. yr bp) (Van der Valk, 1995), especially in the beach plains in between the beach barriers. This clay was predominantly deposited north of the Old Rhine estuary and river channel, which was also migrating northwards around that time. The enhanced rate of clay trapping starting from 2,600 cal. yr bp is attributed to the decrease in mean river discharge, which enabled tides and storm surges to reach further inland, increasing deposition of marine clays (Van der Valk, 1995). In addition, natural and human‐induced peat subsidence may have enhanced the expansion of clay deposition. From *∼*2,500 cal. yr bp onwards, increased habitation and the known practice of de‐watering of peat lands to increase habitability led to sea ingression in many back‐barrier areas along the Dutch coast (Pierik, Cohen, et al., 2017; Vos, 2015). Local peat reclamations in the areas between and immediately behind the beach barriers may therefore have sequentially led to subsidence, enlarging accommodation space and permitting sea ingression. Finally, although the mean annual discharge and fluvial sand transport strongly decreased during this period, river floods still transported ample clay to the Old Rhine estuary (Van Dinter et al., 2017). Following the decrease in fluvial discharge, the coastal promontory and beach barriers were reworked by waves. Similar reworking of abandoned promontory systems (Bhattacharya & Giosan, 2003) has been reconstructed for the previous Rhine outlet near Rotterdam (Hijma & Cohen, 2011b), the Po delta (Correggiari, Cattaneo, & Trincardi, 2005) and the Usumacinta‐Grijalva beach‐barrier plain (Nooren et al., 2017). The estuary mouth had a more sinuous shape during the final stages of its existence, as can be seen on historical maps and the present‐day topography (Figures 2a and 10: 2,000 cal. yr bp). This shape was induced by the northward littoral drift becoming more important relative to river discharge. The Old Rhine estuary is not the only system developing such a sinuous channel in its final stages. For example, the Oer‐IJ estuary, located 40 km northwards along the Dutch coast, developed a similar shape during its final stages of existence, as can be expected from the similar coastal orientation and dominant long‐shore transport direction (Vos, de Koning, & van Eerden, 2015). In addition, Rodríguez‐Ramírez et al. (1996, 2014) found a similar sinuous shape for the Guadalquivir estuary in Spain. This system had a small river influx and, similar to the Old Rhine, only developed its sinuous shape after the river discharge decreased.

From Roman times onwards and throughout the first millennium ad, the Old Rhine functioned as one of the main shipping routes between the hinterland and the North Sea (Jansma, Van Lanen, & Pierik, 2017; Van Lanen, Jansma, van Doesburg, & Groenewoudt, 2016). Storm surges from time to time affected the Old Rhine estuary, and occasionally dune sand covered parts of the estuary floodplain, notably in the 10th century (Van der Valk, 1995). Construction of a river dam near Wijk bij Duurstede in 1122 ad effectively sealed off the already nearly silted‐up downstream Old Rhine from river water, which implies that the Old Rhine distributary and its surrounding landscape from that moment on functioned as a local drainage basin. The youngest clay deposits in the Old Rhine estuary also date to the 12th century ad. These had a marine origin as shown by the occurrence of estuarine shell specimens in life position in the sediments (Heeringen & van der Valk, 1989). The Old Rhine to sea connection disappeared entirely around 1300 ad. This was probably the effect of substantial loss of the river‐induced enhanced ebb flows and of the accompanying progressive reduction of the tidal prism, which enabled coastal long‐shore drift to clog the sea entrance together with dune drift sands (De Haas et al., 2018; Van der Valk, 1995, 2011).

### 5.4 Assessment of effects of changing boundary conditions

**Figure 11:**
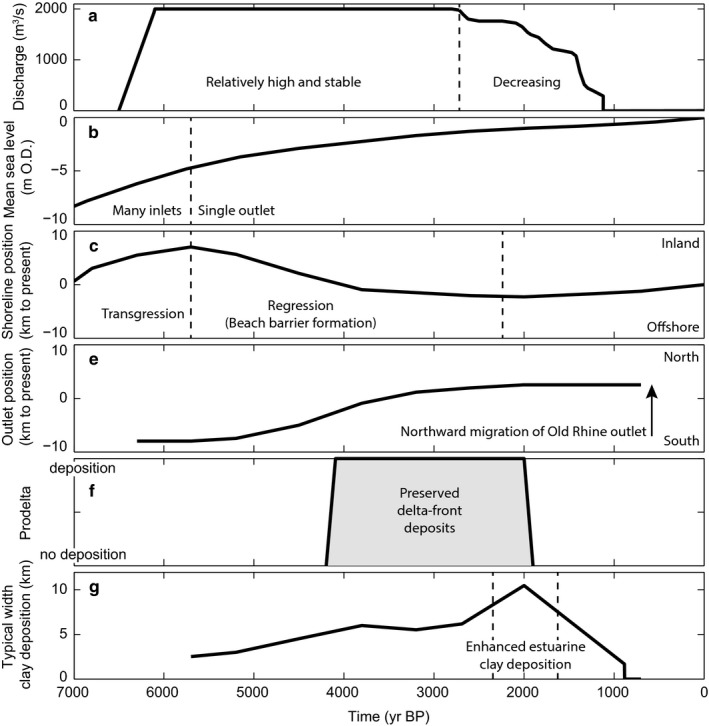
Summary of boundary conditions and the main developments of the Old Rhine estuary and its surrounding area. All ages in cal. yr bp

Figure 11a,b summarizes and compares the timing of the most important boundary conditions, with the main developments of the Old Rhine estuary. The transition between coastal transgression (barrier back‐stepping) to coastal regression (barrier progradation) (Figure 11c) was caused by closure of most inlets along the coast from *∼*5,700 cal. yr bp onwards (Figure 11b). The period of beach‐barrier progradation that commenced around 5,700 cal. yr bp lasted for at least 3,000 yrs. Yet, beach‐barrier progradation was already slowing down between *∼*3,800 and 3,000 cal. yr bp along the Old Rhine estuary (Figure 8), eventually ceasing between 2,500 and 2,000 cal. yr bp (Cleveringa, 2000; Roep et al., 1991; Van der Valk, 1995). Although this roughly coincides with the substantial decrease of Old Rhine discharge, the consistency in the ending of progradation along the entire coast of the western Netherlands (Beets & Van der Spek, 2000; Roep et al., 1991) suggests that coastal progradation had a largely marine rather than fluvial cause. Nevertheless, around the Old Rhine outlet progradation was locally enhanced due to fluvial sediment supply, as suggested by the seaward‐curving beach barriers. The northward migration of the inlet occurred well before the discharge started to decrease suggesting that this migration and the formation of the spit in front of the estuary were the result of marine processes, possibly changes in the wave climate or sand supply from the south. The fastest northward migration of the mouth along the coast coincides with a period of fast regression around 4,000 cal. yr bp, when relatively greater quantities of sand were available to reshape the estuary mouth morphology.

The Old Rhine estuary closed off after river discharge ceased. This shows that substantial fluvial input was a prerequisite for the Old Rhine estuary to persist over time, and waning fluvial input led to reduction of the former quasi‐equilibrium dimensions of the estuary resulting from sediment input equalling sediment output. This is consistent with the general evolution of tidal systems along the western parts of the Dutch coast where tidal systems tend to fill up and close off in the absence of substantial fluvial input and creation of accommodation by rapid sea‐level rise (De Haas et al., 2018; Van der Spek, 1996). Van den Berg, Jeuken, and Van der Spek (1996) and De Haas et al. (2018) hypothesized that the final stages of estuary infilling are mainly the result of mud trapping and expansion of vegetation into the estuary, which further enhances mud trapping, inducing a positive feedback mechanism that ultimately leads to closure of an estuary. The reconstructed enhanced mud trapping in the final stages of the Old Rhine estuary as a result of loss of river discharge supports this hypothesis.

The data presented here do not allow the planform shape and dimensions of the estuary to be determined before 2,500 cal. yr bp due to the continuous northward and seaward migration of its channels. It is thus unknown whether the estuary was able to attain a planform shape similar to an ‘ideal’, trumpet‐shaped, estuary (cf. Savenije, 2005). A further unknown is how planform shape and size came about in the aggradational system of the Old Rhine estuary, where the sedimentation of levees probably self‐confined the estuary despite sea‐level rise. Future numerical and physical modelling of estuaries, using well‐constrained initial and boundary conditions from geological reconstructions, may help unravel the processes that are key to estuary shape formation in wide aggrading basins.

## 6 CONCLUSIONS

The long‐term palaeogeographical evolution and regional boundary conditions of the former Old Rhine estuary, which was active on the Dutch coast from *∼*6,500 cal. yr bp to *∼*1,000 cal. yr bp, was synthesized based on a rich geological dataset and literature.

The Old Rhine River formed around 6,500 cal. yr bp by a northward avulsion near the apex of the Rhine delta. By 6,100 cal. yr bp, the newly formed distributary conveyed most of the Rhine discharge. Initially, the Old Rhine entered an extensive back‐barrier basin, where it connected to and followed a tidal channel to the open sea. As a result of a decelerating rate of sea‐level rise and ample sedimentation, the back‐barrier basin silted up and large parts of the barrier coast progressively closed except for the Old Rhine outlet. At that time the Old Rhine outlet had established itself as an estuary, which traversed the further closed‐off tidal basin. The estuary was laterally confined by levee formation and the formation of vast peatlands behind these levees. This confinement made offshore and fluvial sediment, which was formerly transported into the back‐barrier basins, available for up to 9 km of coastal progradation from 5,700 cal. yr bp to *∼*2,000 cal. yr bp through accretion of a beach‐barrier complex. During this period, the Old Rhine estuary mouth migrated northwards by *∼*10 km from its initial position, forced by littoral drift. From *∼*3,000 cal. yr bp onwards, and most significantly from 2,200 to 1,500 cal. yr bp, a series of upstream avulsions in the Rhine River network caused redirection of the majority of the Rhine River discharge into the North Sea through another outlet in the south, so that the discharge of the Old Rhine strongly reduced. The reduced fluvial input had a significant effect on the Old Rhine estuary; it allowed for a period of marine clay deposition within the estuary and flanking low‐lying areas. Finally, the strong reduction in the fresh water discharge culminated in the silting up of the Old Rhine estuary, ultimately resulting in its closure around 1200 ad.

The results highlight that estuarine evolution can strongly depend on the interaction with the regional landscape, and show that fluvial input is essential for tidal system dynamics and ultimately, for its survival. This reconstruction provides a dataset for the validation of numerical and physical modelling of high‐stand estuaries forming in wide and shallow back‐barrier basins.

## CONFLICT OF INTEREST

The authors declare no conflicts of interest.
