# Supplementary material for: Long‐term evolution of the Old Rhine estuary: Unravelling effects of changing boundary conditions and inherited landscape
Source: Depos Rec. 2019 Jan 2;5(1):84–108. doi: 10.1002/dep2.56 (PMC6743690; doi:10.1002/dep2.56)
Supplement: Supplementary file 1 [file DEP2-5-84-s001.pdf]

Supplementary Tables

Table 1: Organic radiocarbon dates in the study area down to -20 m O.D. Data has been calibrated with OxCal 4.3 (Ramsey, 2009) using the IntCal13-curve (Reimer et al., 2013). Coordinates are in the Dutch national triangulation system. Dates for which no references given are previously unpublished.

Lab code (UCIAMS)	X	Y	Sample Depth (m NAP)	Age (14C yr BP)	δ13C (p.mil.)	Age $\pm$ 2σ (cal. yr BP)	Material dated	Reference
GrN-01619	104000	456000	-10.5	7090$\pm$ 100		7910 $\pm$ 200	Peat	Jelgersma (1961)
GrN-01622	104000	456000	-5.02	4880$\pm$ 80		5620 $\pm$ 200	Peat	Jelgersma (1961)
GrN-01617	102000	469000	-10.92	6475$\pm$ 70		7380 $\pm$ 120		Jelgersma (1961); Zagwijn (1961)
GrN-01636	102000	469000	-11.3	8165$\pm$ 105		9130 $\pm$ 320		Zagwijn (1961)
GrN-01618	102000	469000	-11.36	8700$\pm$ 110		9760 $\pm$ 340		Jelgersma (1961); Zagwijn (1961)
GrN-02125	103000	469000	-3.68	1610$\pm$ 50		1490 $\pm$ 120		Zagwijn (1961)
	103000	469000	-5.08	4275$\pm$ 45		4840 $\pm$ 140		Zagwijn (1960)
GrN-01569	96750	476150	-1.67	3470$\pm$ 60		3740 $\pm$ 160	Peat	Jelgersma (1961)
GrN-01623	92500	466500	-2.5	4240$\pm$ 75		4760 $\pm$ 220	Peat	Jelgersma (1961)
GrN-03552	100000	477000	-3.69	4300$\pm$ 80		4890 $\pm$ 280		Zagwijn and De Jong (1965)
GrN-03563	100000	477000	-4.57	4820$\pm$ 100		5540 $\pm$ 240		Zagwijn and De Jong (1965)
GrN-01621	104000	456000	-9.94	6390$\pm$ 85		7320 $\pm$ 160	Peat	Jelgersma (1961)
GrN-00792	95575	470650	-12.73	7970$\pm$ 60		8830 $\pm$ 220	Phragmites Peat	Jelgersma (1961); Zagwijn (1961)
UtC-14221	95193	462131	-7.38	7090$\pm$ 90	-29.3	7910 $\pm$ 180	Basal peat	Hijma et al. (2009)
UtC-14222	95752	462491	-12.71	6840$\pm$ 70	-29.9	7690 $\pm$ 140	Basal peat	Hijma et al. (2009)
UtC-14223	96800	462988	-12.12	6840$\pm$ 60	-29.1	7680 $\pm$ 120	Basal peat	Hijma et al. (2009)
UtC-15337	94862	459157	-12.49	6910$\pm$ 60	-25.6	7750 $\pm$ 120	Wood peat	Hijma et al. (2009)
GrN-12111	87560	469650	0.52	1530$\pm$ 25	-28.3	1430 $\pm$ 100		
Poz-42559	77530	455210	0.62	2980$\pm$ 30		3150 $\pm$ 100	Macrofossils	Van Beurden (2011)
Poz-42561	77530	455210	0.78	2705$\pm$ 30		2810 $\pm$ 60	Macrofossils	Van Beurden (2011)
Poz-42560	77530	455210	0.49	2935$\pm$ 35		3090 $\pm$ 120	Macrofossils	Van Beurden (2011)
UtC-15336	94862	459157	-9.46	5800$\pm$ 50	-24.7	6600 $\pm$ 120	Fen-wood peat	Hijma et al. (2009)
GrN-08421	89725	462575	-1.75	3395$\pm$ 25	-28.2	3640 $\pm$ 80		Van de Plassche (1982)
GrN-08422	89725	462575	-1.7	3305$\pm$ 50	-27.9	3530 $\pm$ 120		Van de Plassche (1982)
GrN-08423	89725	462575	-1.55	3045$\pm$ 55	-27.7	3240 $\pm$ 160		Van de Plassche (1982)
GrN-15010	81700	457700	-0.05	3320$\pm$ 35		3550 $\pm$ 100		
GrN-15012	80950	454900	0.2	3310$\pm$ 35		3530 $\pm$ 100		
GrN-11310	93720	462620	-1.88	3420$\pm$ 55		3680 $\pm$ 160	Very clayey peat	De Jong (1983)
GrN-09395	89890	465190	-0.86	3030$\pm$ 55		3230 $\pm$ 160	Humic clay, small roots removed	De Jong (1981)
GrN-08418	89600	462600	-1.59	3565$\pm$ 40		3860 $\pm$ 120	Clayey wood peat	Van de Plassche (1982)
GrN-08419	89600	462600	-1.64	3425$\pm$ 70		3690 $\pm$ 180	Clayey organic material	Van de Plassche (1982)
GrN-08420	89600	462600	-1.98	3615$\pm$ 40		3930 $\pm$ 120	Clayey Carex-Phragmnites peat	Van de Plassche (1982)
GrN-08731	95690	465580	-4.93	4690$\pm$ 70		5430 $\pm$ 180	Very humic clay, thin layer	Bosch and Pruissers (1978)

GrN-08862	87750	457900	-2.69	4240± 140	4800 ± 420	Phragmites peat	Van de Plassche (1982)
GrN-08863	87750	457900	-1.96	4000± 60	4480 ± 200	Fen-wood peat	Van de Plassche (1982)
GrN-08864	87750	457900	-1.33	3770± 45	4140 ± 160	Fen-wood peat	Van de Plassche (1982)
GrN-08865	87750	457900	-1.04	3860± 50	4280 ± 160	Fen-wood peat	Van de Plassche (1982)
GrN-08866	87750	457900	-1.04	3470± 60	3740 ± 160	Fen-wood peat	Van de Plassche (1982)
GrN-08907	86850	463325	-0.9	3325± 30	3550 ± 80	Sedge peat, sandy	Van de Plassche (1982)
GrN-08908	87150	463400	-0.99	2710± 30	2810 ± 60	Clayey Phragmites-Carex peat	Van de Plassche (1982)
GrN-08909	87500	462500	-1.64	3495± 55	3770 ± 140	Slightly clayey Sedge peat	Van de Plassche (1982)
GrN-08910	89350	463200	-1.47	3515± 55	3790 ± 140	Slightly clayey fen-woord peat	Van de Plassche (1982)
GrN-08911	88100	462400	-1.46	3245± 50	3480 ± 120	Clayey Sedge peat	Van de Plassche (1982)
GrN-08084	97990	459730	-3.34	3435± 35	3700 ± 120	Wood peat	Bosch and Pruissers (1978)
GrN-08085	97990	459730	-4.14	3730± 60	4080 ± 180	Phragmites peat, thin layer	Bosch and Pruissers (1978)
GrN-08086	97990	459730	-4.42	4250± 60	4780 ± 200	Clayey Phragmites peat, thin layer	Bosch and Pruissers (1978)
GrN-08087	97990	459730	-4.74	4350± 60	4950 ± 200	Clayey Phragmites peat, of thin layer	Bosch and Pruissers (1978)
GrN-08088	97990	459730	-4.99	4780± 60	5500 ± 160	Clayey Phragmites peat	Bosch and Pruissers (1978)
GrN-08093	98150	460450	-3.64	4405± 40	5000 ± 200	Clayey peat	Bosch and Pruissers (1978)
GrN-08163	95690	465580	-2.56	2625± 50	2750 ± 140	Clayey Phragmites peat	Bosch and Pruissers (1978)
GrN-08164	95690	465580	-3.5	3820± 30	4220 ± 120	Phragmites peat, thin layer	Bosch and Pruissers (1978)
GrN-08165	95690	465580	-3.79	4110± 60	4650 ± 200	Phragmites peat	Bosch and Pruissers (1978)
GrN-08166	95690	465580	-3.92	4260± 60	4800 ± 200	Phragmites peat	Bosch and Pruissers (1978)
GrN-08167	95690	465580	-5.23	4560± 60	5210 ± 240	Phragmites peat	Bosch and Pruissers (1978)
GrN-08411	89000	463000	-1.56	4140± 40	4680 ± 160	Shell doublets (Cerastoderma)	Van de Plassche (1982)
GrN-08412	90500	460800	-2.1	4245± 35	4800 ± 120	Sandy wood peat	Van de Plassche (1982)
GrN-08413	90500	460800	-1.84	4070± 55	4600 ± 220	Wood peat	Van de Plassche (1982)
GrN-08414	89500	462500	-0.61	2955± 50	3110 ± 160	Sandy wood peat	Van de Plassche (1982)
GrN-08415	89500	462500	-0.93	2985± 35	3160 ± 120	Sandy wood peat	Van de Plassche (1982)
GrN-08416	89500	462500	-1.27	3135± 35	3350 ± 100	Sandy wood peat	Van de Plassche (1982)
GrN-08417	89600	462600	-1.26	3315± 25	3540 ± 80	Fen-wood peat	Van de Plassche (1982)
GrN-01574	82975	461300	-0.19	1680± 55	1590 ± 140	Carex peat	
GrN-01575	82975	461300	0.49	2690± 60	2810 ± 100	Sandy carex peat	
GrN-01620	102550	469775	-9	6320± 70	7250 ± 180	Clayey Phragmites peat	
GrN-01661	95575	470650	-6.34	4780± 60	5500 ± 160	Phragmites peat	
GrN-08914	104250	457750	-10.65	7035± 45	7870 ± 100	Wood peat	Van de Plassche (1982)
GrN-08915	99475	457375	-11.51	7020± 70	7850 ± 140	Reed peat	Van de Plassche (1982)
GrN-08926	88100	462400	-1.18	2985± 30	3160 ± 120	Clayey sedge peat	Van de Plassche (1982)
GrN-09396	89890	465190	-1.07	2850± 35	2960 ± 120	Clayey gyttja	De Jong (1981)

GrN-09397	89890	465190	-1.75	3330± 35	3560 ± 100	Slightly clayey gyttja	De Jong (1981)
GrN-09428	89135	454475	-5.03	4400± 40	4990 ± 200	Phragmites peat	De Mulder et al. (1983)
GrN-09429	89135	454475	-5.97	5045± 40	5800 ± 120	Gyttja with many reed remains	De Mulder et al. (1983)
GrN-09807	89135	454475	-6.98	5625± 45	6400 ± 100	Phragmites peat	De Mulder et al. (1983)
GrN-09808	89135	454475	-11.28	6345± 40	7280 ± 120	Gyttja with wood / reed remains	De Mulder et al. (1983)
GrN-10237	97090	466500	-4.1	4045± 40	4540 ± 180	Phragmites peat	De Mulder et al. (1983)
GrN-10238	97090	466500	-4.45	4640± 40	5390 ± 120	Slightly clayey Phragmites peat	De Mulder et al. (1983)
GrN-10239	97090	466500	-7.58	5660± 40	6440 ± 100	Slightly clayey Phragmites peat	De Mulder et al. (1983)
GrN-10240	97090	466500	-9.08	6310± 45	7240 ± 100	Strongly clayey Phragmites peat	De Mulder et al. (1983)
GrN-10241	97090	466500	-11.82	6900± 50	7740 ± 100	Phragmites peat	De Mulder et al. (1983)
GrN-10947	80310	457180	2.41	1030± 35	950 ± 80	Strongly sandy peat	
GrN-10948	80310	457180	2.3	850± 20	760 ± 40	Sandy peat	
GrN-10949	80310	457180	1.98	1135± 25	1040 ± 100	Sandy peat	
GrN-10950	80310	457180	1.9	1455± 30	1340 ± 60	Slightly sandy peat	
GrN-10951	80310	457180	1.63	1905± 30	1850 ± 80	Slightly sandy peat, wood remains	
GrN-10952	80310	457180	2.76	1125± 30	1030 ± 100	Strongly humic sand	
GrN-11695	101110	458615	-4.53	4585± 40	5270 ± 240	Phragmites peat	
GrN-11696	101095	458512	-3.83	4045± 35	4530 ± 160	Base of wood peat	
GrN-12088	82340	458450	1.62	1090± 45	1010 ± 100	Sandy peat	
GrN-12089	80310	457180	2.6	905± 40	830 ± 100	Strongly humic sand	
GrN-12105	82340	458450	1.51	1130± 35	1040 ± 100	Slightly sandy peat, basal	
GrN-12106	80310	457180	2.48	1035± 40	950 ± 100	Strongly sandy peat	
GrN-12107	80310	457180	2.14	1020± 30	940 ± 60	Sandy peat	
GrN-15121	84810	462130	-14.52	4830± 60	5550 ± 160		Cleveringa (2000)
GrN-15424	100650	458600	-0.8	3505± 35	3770 ± 100	Wood	
GrN-15425	100650	458600	-1.5	3840± 40	4260 ± 160	Wood	
GrN-15426	100650	458600	-2.1	4110± 35	4650 ± 180	Wood	
GrN-15427	100650	458600	-2.64	4130± 350	4660 ± 940	Wood	
GrN-24095	105300	460200	0	1954± 14	1900 ± 40	Wood	
GrN-24096	105300	460200	0	1945± 17	1890 ± 40	Wood	
GrN-24097	105300	460200	0	1958± 15	1910 ± 40	Wood	
GrN-24098	105300	460200	0	1945± 20	1890 ± 60	Wood	
GrN-24099	105300	460200	0	1959± 18	1910 ± 40	Wood	
UtC-14448	89714	458148	-10.18	6430± 59	7350 ± 100	Phragmites Peat	
UtC-14449	89714	458148	-8.51	5701± 69	6500 ± 160	Phragmites Peat	
UtC-14450	89714	458148	-4.93	4561± 63	5210 ± 240	Phragmites Peat	

UtC-10758	90234	463476	-16.2	6689± 49	-27.3	7560 ± 80	TM and charcoal (peat)	Busschers et al. (2005)
GrA-49579	86795	459338	-15.02	7095± 40		7920 ± 80	Macrofossils	
GrA-49530	86795	459338	-15.54	7350± 45		8160 ± 160	Macrofossils from basal peat	
UtC-14457	91762	457067	-9.6	6210± 60		7110 ± 160	Organic layer with transition to peat	
UtC-14458	91762	457067	-9.36	6030± 60		6880 ± 180	Phragmites Peat	

Table 2: Shells radiocarbon dates in the study area down to -20 m O.D. Data has been calibrated with OxCal 4.3 (Ramsey, 2009) using the Marine13-curve (Reimer et al., 2013). Coordinates are in the Dutch national triangulation system. Dates for which no references given are previously unpublished.

Lab code (UCIAMS)	X	Y	Sample Depth (m NAP)	Age (14C yr BP)	δ13C (p.mil.)	Age $\pm$ 2σ (cal. yr BP)	Material dated	Reference
GrN-16187	89700	460240	-3.6	4710 ± 60	-6.08	5450 ± 160	*Scrobicularia plana*, in viva	Van der Valk (1995)
GrN-12747	80035	458870	-11.15	3910 ± 150	-1.6	4350 ± 440	*Spisula subtruncata*, loose shells	Van der Valk (1995)
GrN-12746	80035	458870	-4.15	3510 ± 70	-1.1	3790 ± 180	*Spisula subtruncata*, loose shells	Van der Valk (1995)
UtC-14440	89714	458148	-8.4	5568 ± 60	-5.5	6360 ± 100	*Cerast.*, one valve	
UtC-14442	89714	458148	-8.09	5508 ± 60	-5.5	6310 ± 120	*Scrobi.*, one valve	
UtC-14467	93732	456192	-6.35	5538 ± 50	-5	6340 ± 80	*Scrobi.*, one valve	
UtC-13009	77940	463641	-16.34	5746 ± 38	-3.1	6550 ± 100	*Spisula*, juveniles	
UtC-14466	93732	456192	-7.89	5618 ± 60	-5.9	6400 ± 120	*Scrobi.*, one valve	
UtC-14446	89714	458148	-7.07	5418 ± 80	-6.8	6190 ± 200	*Scrobi.*, double valve	
UtC-14444	89714	458148	-7.6	5518 ± 60	-5.8	6320 ± 120	*Scrobi.*, one valve	
UtC-14445	89714	458148	-7.3	5628 ± 100	-5.7	6430 ± 220	*Scrobi.*, double valve	
UtC-14443	89714	458148	-7.78	5558 ± 60	-5.9	6360 ± 100	*Scrobi.*, one valve	
GrN-19250	79800	458990	-0.79	3200 ± 90	-1.33	3420 ± 220	*Mytilisedulis*, couplet	Van der Valk (1995)
UtC-14465	93732	456192	-8.28	5588 ± 50	-4.5	6370 ± 100	*C.edule*	
UtC-14456	91762	457067	-6.1	5168 ± 60	-6	5920 ± 180	*Scrobi.*	
UtC-14447	89714	458148	-6.61	5278 ± 60	-7.4	6070 ± 160	*Scrobi.*, double valve	
UtC-14469	93732	456192	-5.71	5328 ± 50	-5.4	6110 ± 160	*Scrobi.*, double valve	
UtC-14460	93732	456192	-9.44	5538 ± 80	-5.1	6340 ± 160	*C.edule*, double valve	
UtC-14459	93732	456192	-9.52	5508 ± 80	-5.6	6310 ± 180	*C.edule*, one valve	
UtC-14461	93732	456192	-9.43	5638 ± 60	-4.5	6420 ± 140	*Glaucum*, double valve	
GrN-12748	80430	458870	-18.2	6240 ± 90	-3.89	7140 ± 220	*Mytilus edulis*, double valve	Van der Valk (1996)
UtC-8197	84190	463040	-5.94	3672 ± 43	-2.3	4010 ± 140	*Spisula*, juvenile	Cleveringa (2000)
UtC-8194	84190	463040	-2.64	3599 ± 34	-1.1	3910 ± 100	*Spisula*, juvenile	Cleveringa (2000)
UtC-8203	84190	463040	-16.49	5646 ± 44	-3.1	6420 ± 100	*Spisula*, juvenile, blue, periostraucum	Cleveringa (2000)

UtC-8198	84190	463040	-7.66	3676 ± 47	-1.2	4010 ± 140	*Spisula*, juvenile, blue, some periostraucum	Cleveringa (2000)
UtC-8196	84190	463040	-5.06	3620 ± 37	-2	3940 ± 120	*Spisula*, juvenile, periostraucum	Cleveringa (2000)
UtC-8202	84190	463040	-13.71	4849 ± 40	-2.7	5580 ± 100	*Spisula*, juvenile, periostraucum	Cleveringa (2000)
UtC-8199	84190	463040	-8.88	3633 ± 37	-1.1	3950 ± 120	*Spisula*, juvenile, slightly blue, periostraucum	Cleveringa (2000)
UtC-8201	84190	463040	-12.7	4593 ± 42	-2	5300 ± 240	*Spisula*, juveniles (three)	Cleveringa (2000)
UtC-8200	84190	463040	-11.93	3835 ± 37	-2.5	4250 ± 160	*Spisula*, juveniles (two), blue, some periostraucum	Cleveringa (2000)
UtC-8195	84190	463040	-3.95	3672 ± 43	-1.1	4010 ± 140	*Spisula*, juveniles (two), periostraucum	Cleveringa (2000)
UtC-8213	83970	463520	-16.69	5843 ± 42	-4.4	6650 ± 120	*Macoma*, fully grown	Cleveringa (2000)
UtC-8207	83970	463520	-7.77	3669 ± 35	-1.9	4000 ± 120	*Spisula Elliptica*, juvenile, periostraucum	Cleveringa (2000)
UtC-8205	83970	463520	-4.79	3619 ± 41	-1.2	3940 ± 120	*Spisula*, juvenile, periostracum	Cleveringa (2000)
UtC-8210	83970	463520	-11.87	4280 ± 45	-1.9	4850 ± 120	*Spisula*, juvenile, periostracum	Cleveringa (2000)
UtC-8211	83970	463520	-12.67	4688 ± 70	-1.4	5430 ± 180	*Spisula*, juvenile, slightly damaged	Cleveringa (2000)
UtC-8204	83970	463520	-3.74	3538 ± 60	-0.9	3820 ± 160	*Spisula*, juvenile, slightly weathered	Cleveringa (2000)
UtC-8206	83970	463520	-5.16	3663 ± 45	-1.2	4000 ± 140	*Spisula*, juvenile, some periostracum	Cleveringa (2000)
UtC-8209	83970	463520	-9.86	3979 ± 44	-1.5	4450 ± 140	*Spisula*, juvenile, some periostraucum	Cleveringa (2000)
UtC-14468	93732	456192	-6	5398 ± 50	-5.7	6190 ± 160	*Scrobi.*, one valve	
UtC-13010	77940	463641	-18.61	6981 ± 40	-4.6	7820 ± 120	*Mytilusedulis*, fragment	
UtC-13008	75932	460573	-19.55	7345 ± 42	-8.3	8150 ± 140	*Cerast.*	
UtC-13011	77940	463641	-19.99	6913 ± 42	-5.4	7750 ± 100	*Cerast.*, juvenile	
UtC-14451	91762	457067	-8.12	5309 ± 69	-10.6	6090 ± 180	*Cerast.*, double valve	
UtC-14462	93732	456192	-9.25	5628 ± 59	-4.8	6410 ± 140	*Glaucum*, one valve	
UtC-14452	91762	457067	-7.45	5533 ± 56	-5.9	6340 ± 100	*Scrobi.*, double valve	
UtC-14463	93732	456192	-9.06	5578 ± 60	-4.9	6370 ± 100	*C.edule*, double valve	

UtC-14464	93732	456192	-8.76	5618 ± 50	-3.6	6390 ± 100	*Glaucum*, double valve	
GrN-19425	79800	458990	-0.79	3260 ± 30		3490 ± 80	*Mytilus edulis*	Van der Valk (1995)
UtC-14453	91762	457067	-7.07	5464 ± 60	-6.8	6260 ± 140	*Scrobi.*, one valve	
UtC-14454	91762	457067	-6.67	5098 ± 90	-7.2	5840 ± 220	*C.edule*, double valve	
UtC-14455	91762	457067	-6.22	5228 ± 60	-5.2	6020 ± 180	*Scrobi.*, one valve	
UtC-14441	89714	458148	-8.2	5548 ± 60	-4.8	6350 ± 100	*Scrobi.*, one valve	
UtC-13007	75932	460573	-15.48	4829 ± 39	-2.3	5550 ± 100	*Spisula*, double valve	
GrN-12147	81250	456650	-5.92	4200 ± 50	-1.26	4720 ± 160	*Cardium Edule*, double valve	
GrA-03424	95650	471275	-10.01	5390 ± 50	-8.49	6180 ± 160	*Cerast. glauc.*, juvenile	
GrA-03645	95650	471275	-7.21	5320 ± 60	-4.49	6100 ± 160	*Macoma balthica*	
GrA-03642	95650	471275	-8.04	5350 ± 60	-4.6	6130 ± 160	*Scrobi. plana*	
UtC-04529	95650	471275	-4.79	4838 ± 50	-5.59	5560 ± 120	*Scrobi. plana*	
GrA-03425	95650	471275	-8.86	5500 ± 50	-5.21	6300 ± 100	*Scrobi. plana*	
GrA-03426	95650	471275	-6.64	5230 ± 50	-6.59	6010 ± 160	*Scrobi. plana*	
GrN-11634	81625	455250	-7.65	4750 ± 80		5470 ± 180	*Spisula*, double valve	De Mulder et al. (1983)
GrA-03412	95650	471275	-4.93	5250 ± 70	-2.73	6040 ± 180	*Spisula subtruncata*	
GrA-03709	87920	457730	-10.11	5220 ± 60	-2.81	6010 ± 180	*Spisula subtruncata*	
UtC-15527	80699	458702	-18.22	6268 ± 70	-4.3	7180 ± 180	*Mytilus*	
UtC-15528	80699	458702	-17.08	6128 ± 50	-6.6	7030 ± 160	*Mytilus*, couplet	
UtC-15529	80699	458702	-15.25	4828 ± 70	-3	5550 ± 180	*Spisula*, juvenile	
UtC-15530	80699	458702	-13.25	4098 ± 60	-1.7	4640 ± 220	*Spisula*, couplet, juvenile	
GrN-11987	86000	461850	-4.14	3920 ± 100		4360 ± 300	*Spisula subtruncata*	Van der Valk (1995)
GrN-11988	86000	461850	-11.14	4525 ± 90		5170 ± 280	*Spisula subtruncata*	Van der Valk (1995)
GrN-11989	86400	461785	-6.48	4490 ± 105		5140 ± 300	*Spisula subtruncata*	Van der Valk (1995)
GrN-11990	86400	461785	-10.1	4730 ± 105		5450 ± 260	*Spisula subtruncata*	Van der Valk (1995)
GrN-11991	86710	461370	-6.4	4300 ± 110		4890 ± 360	*Spisula subtruncata*	Van der Valk (1995)
GrN-11993	87360	461070	-9.39	4520 ± 110		5170 ± 320	*Spisula subtruncata*	Van der Valk (1995)
GrN-11992	87360	461070	-6.69	4410 ± 105		5060 ± 300	*Spisula subtruncata*	Van der Valk (1995)
GrN-15120	87390	460500	-6.92	4630 ± 93	-2.25	5330 ± 300	*Spisula subtruncata*, fragments	Van der Valk (1995)
GrN-08411	89000	463075	-1.56	4140 ± 37	-1.5	4690 ± 160	*Cerastoderma*, double valve	Van der Valk (1995)

References

Bosch, J. H. A., Pruissers, A. P., 1978. Unpublished technical report. Tech. rep., Geological Survey of The Netherlands.

Busschers, F., Weerts, H., Wallinga, J., Cleveringa, P., Kasse, C., De Wolf, H., Cohen, K., 2005. Sedimentary architecture and optical dating of Middle and Late Pleistocene Rhine-Meuse deposits-fluvial response to climate change, sea-level fluctuation and glaciation. Netherlands Journal of Geosciences 84 (01), 25–41.

Cleveringa, J., 2000. Reconstruction and modelling of Holocene coastal evolution of the western Netherlands. Ph.D. thesis, Universiteit Utrecht.

De Jong, J., 1981. Unpublished technical report. Tech. rep., Geological Survey of The Netherlands.

De Jong, J., 1983. Unpublished technical report. Tech. rep., Geological Survey of The Netherlands.

De Mulder, E. F. J., Pruissers, A. P., Zwaan, H., 1983. Kwartairgeologie van 's-Gravenhage. Tech. Rep. 37, 12-43, Mededelingen Rijks Geologische Dienst 37.

Hijma, M. P., Cohen, K. M., Hoffmann, G., Van der Spek, A. J. F., Stouthamer, E., 2009. From river valley to estuary : the evolution of the Rhine mouth in the early to middle Holocene (western Netherlands, Rhine-Meuse delta). Netherlands Journal of Geosciences 88 (1), 13–53.

Jelgersma, S., 1961. Holocene sea level changes in the Netherlands. Mededelingen Rijks Geologische Stichting 6 (7), 100 pp.

Ramsey, C. B., 2009. Bayesian analysis of radiocarbon dates. Radiocarbon 51 (01), 337–360.

Reimer, P. J., Bard, E., Bayliss, A., Beck, J. W., Blackwell, P. G., Ramsey, C. B., Buck, C. E., Cheng, H., Edwards, R. L., Friedrich, M., et al., 2013. IntCal13 and Marine13 radiocarbon age calibration curves 0–50,000 years cal BP. Radiocarbon 55 (4), 1869–1887.

Van Beurden, L., 2011. Den Haag-Vogelwijk Botanisch onderzoek aan een veenafzetting gedateerd in de Brons- en IJzertijd. Tech. rep., Biax.

Van de Plassche, O., 1982. Sea-level change and water-level movements in the Netherlands during the Holocene. Mededelingen Rijks Geologische Dienst 36 (1), 1–93.

Van der Valk, L., 1995. Toelichting bij de bladen 's-Gravenhage West (30W) en 's-Gravenhage Oost (30O). Tech. rep., Rijks Geologische Dienst, Haarlem.

Van der Valk, L., 1996. Geology and sedimentology of Late Atlantic sandy, wave-dominated deposits near The Hague (South-Holland, the Netherlands): a reconstruction of an early prograding coastal sequence. Mededelingen Rijks Geologische Dienst 57, 201–227.

Zagwijn, W. H., 1960. Unpublished technical report. Tech. rep., Geological Survey of The Netherlands.

Zagwijn, W. H., 1961. Unpublished technical report. Tech. rep., Geological Survey of The Netherlands.

Zagwijn, W. H., De Jong, J., 1965. Unpublished technical report. Tech. rep., Geological Survey of The Netherlands.
